# Errors in Human-Robot Interactions and Their Effects on Robot Learning

**DOI:** 10.3389/frobt.2020.558531

**Published:** 2020-10-15

**Authors:** Su Kyoung Kim, Elsa Andrea Kirchner, Lukas Schloßmüller, Frank Kirchner

**Affiliations:** ^1^Robotics Innovation Center, German Research Center for Artificial Intelligence (DFKI GmbH), Bremen, Germany; ^2^Research Group Robotics, University of Bremen, Bremen, Germany

**Keywords:** human-robot interaction (HRI), error-related potentials (ErrPs), reinforcement learning, robotics, long-term learning, learning with prior knowledge

## Abstract

During human-robot interaction, errors will occur. Hence, understanding the effects of interaction errors and especially the effect of prior knowledge on robot learning performance is relevant to develop appropriate approaches for learning under natural interaction conditions, since future robots will continue to learn based on what they have already learned. In this study, we investigated interaction errors that occurred under two learning conditions, i.e., in the case that the robot learned without prior knowledge (cold-start learning) and in the case that the robot had prior knowledge (warm-start learning). In our human-robot interaction scenario, the robot learns to assign the correct action to a current human intention (gesture). Gestures were not predefined but the robot had to learn their meaning. We used a contextual-bandit approach to maximize the expected payoff by updating (a) the current human intention (gesture) and (b) the current human intrinsic feedback after each action selection of the robot. As an intrinsic evaluation of the robot behavior we used the error-related potential (ErrP) in the human electroencephalogram as reinforcement signal. Either gesture errors (human intentions) can be misinterpreted by incorrectly captured gestures or errors in the ErrP classification (human feedback) can occur. We investigated these two types of interaction errors and their effects on the learning process. Our results show that learning and its online adaptation was successful under both learning conditions (except for one subject in cold-start learning). Furthermore, warm-start learning achieved faster convergence, while cold-start learning was less affected by online changes in the current context.

## 1. Introduction

The “human-in-the-loop” approach, e.g., through human feedback, is an interesting approach to learning in robots. Previous studies have used both explicit and implicit human feedback for robot learning, such as active learning of rewards through the use of human ratings (Daniel et al., 2014) or online generation of rewards through the use of EEG-based human feedback (Iturrate et al., 2015; Kim et al., 2017). The most commonly used EEG components are error-related potentials (ErrPs), which are evoked by the perception of unusual human or robot actions (Falkenstein et al., 2000; Parra et al., 2003; van Schie et al., 2004; Iturrate et al., 2010, 2015; Kim and Kirchner, 2013, 2016; Chavarriaga et al., 2014; Kim et al., 2017, 2020; Salazar-Gomez et al., 2017; Ehrlich and Cheng, 2018, 2019b). Single-trial detections of event-related potentials (ERPs) are possible by using machine learning techniques and signal processing methods (Müller et al., 2004; Lotte et al., 2018), which has been demonstrated in various application areas (review, Zhang et al., 2018). In robot learning, single-trial detections are required for online generation of EEG-based human feedback for each robot's actions. One issue in single-trial EEG detections is to hardly achieve 100% classification accuracy (Kirchner et al., 2013). Another issue is a high subject variability between ErrP classification performance, which is well-known in brain-computer interfaces (BCIs) (Blankertz et al., 2009; Vidaurre and Blankertz, 2010; Ahn and Jun, 2015; Jeunet et al., 2015; Morioka et al., 2015; Ma et al., 2019) and brain imaging (Seghier and Price, 2018; Betzel et al., 2019). A relevant question when using EEG-based human feedback in robot learning is the unknown influence of human-robot interaction on the generation of EEG-based human feedback. Indeed, it has not been systematically investigated how human-robot interactions influence the online generation of EEG-based human feedback in general and especially when several interaction components play together in human-robot interaction or cooperation.

The future cooperation with robots requires an intensive investigation of interaction concepts and learning approaches in robot systems with regard to their applicability in poorly controlled environments, in case of faulty or changing human behavior and when using several interaction options. This is important because it is difficult and very strenuous or even impossible for humans to repeatedly behave identically as a robot can. A good example is the interaction with gestures. There are individual differences even in the choice of gestures, not to mention the fine to great differences in the execution of exactly the same gesture by two different people. Depending on the situation in which a person finds himself, the gestures are also performed differently. The execution of gestures also typically changes over time and depending on the frequency of execution. Often, a person spontaneously thinks of another gesture and executes a different gesture. People can cope well with these changes in the behavior of the human interaction partner. Robots or artificial learning processes have much more problems with this.

A conceivable application is that a robot performs pick-and-place tasks together with a human interaction partner. The task is to sort objects differently depending on current situations determined by human behavior (e.g., human gesture). The robot therefore has no completely fixed predefined task procedure, but does know for example which places are feasible for the robot or the human to reach. On the other hand, the human changes the desired places of objects (selection of the reachable places) depending on current situation or task efficiency. For example, the robot picks up objects and place them in locations that correspond to the current human gesture. After the action selection, the robot receives human feedback on the correctness of action selection (e.g., the robot selects a correct position for placing objects or not) and updates an action strategy based on human feedback. In this way, the robot learns an action that corresponds to the current situation determined by human gesture and also adapts an action strategy depending on online changes of human intention. Two interaction errors can occur here: (a) human gestures, which can be easily changed over time or which can vary between different interaction partners (different people), can be misinterpreted by the robot and (b) human implicit feedback in the form of EEG that can be incorrectly decoded, since a decoder is not perfectly trained. Such online learning and adaptation based on human feedback can be beneficial in unknown situations or unknown environments, e.g., space explorations. In this case, the robot has only a little predefined knowledge about task solution before explorations and can extend knowledge directly by learning from human feedback. Further, it can also be relevant in more pre-defined scenarios, i.e., assembly in production line, to adapt to individual preferences.

In order to develop new interaction concepts and learning procedures that can better deal with such changes in human behavior, we first have to investigate which influence which mistakes have on learning in the robot and which influence misbehavior of the robot has on feedback from humans. In this paper we want to use the example of implicit learning of gesture-action pairs from intrinsic human feedback based on brain activity to investigate the effect of errors in the recognition of EEG signals and gestures on interactive learning.

We investigate interaction errors under two conditions. First, the robot learns with prior knowledge and second, without prior knowledge. Although almost all studies on robot learning assume that the robot has no previous knowledge, this is actually a completely unrealistic situation especially for humans. Humans, like many other animals, almost always learn on the basis of previous knowledge. With our study we want to show that there are differences in the effects of interaction errors depending on whether learning takes place with or without previous knowledge.

### 1.1. Concept of Human-Robot Interaction (HRI)

**Figure 1:**
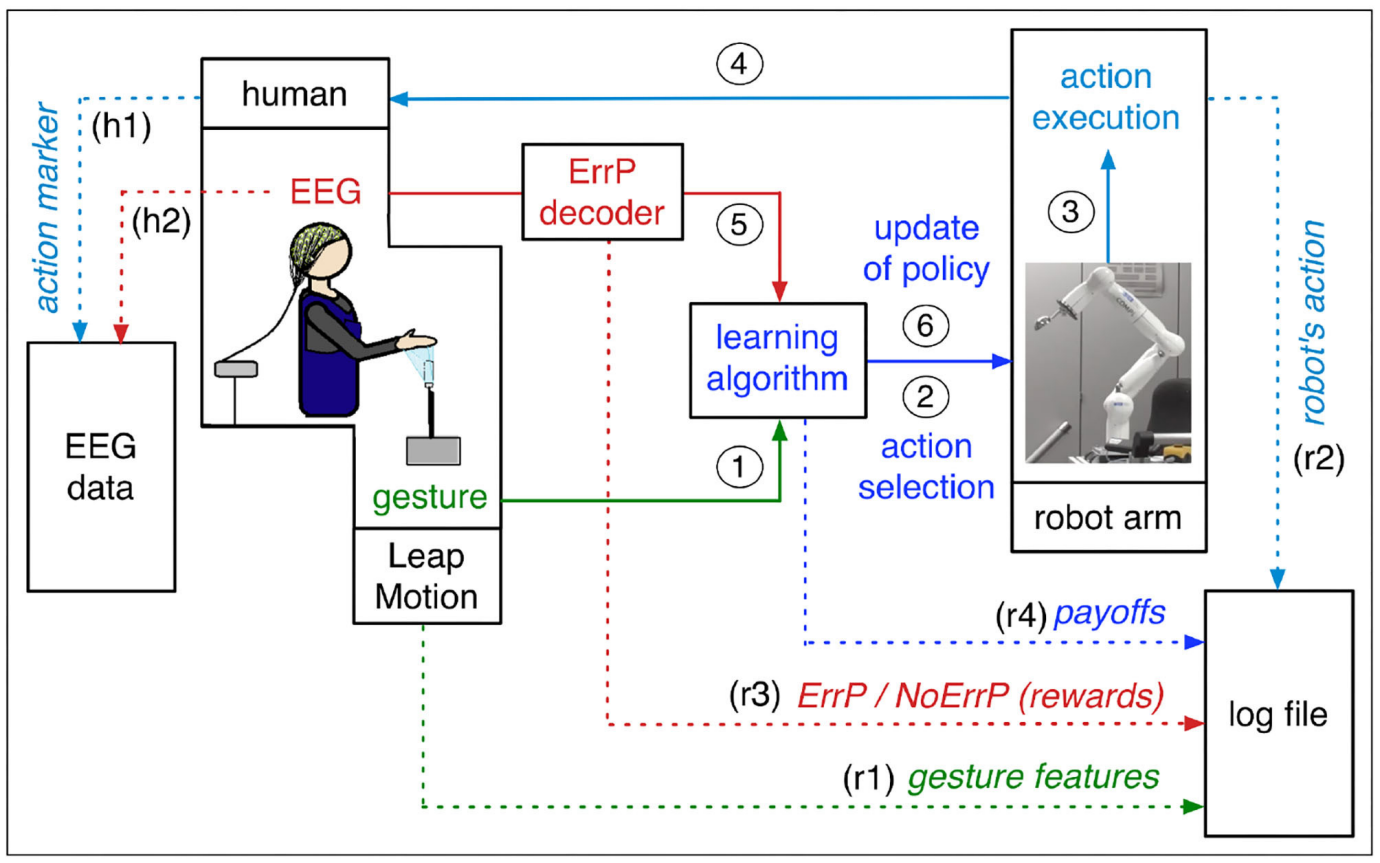
The concept of our approach. Continuous lines represent the information flow of the learning process, and dotted lines represent the logs of the learning process and markers of the EEG data. Solid lines: The subject communicates with the robot in the form of gestures and gesture features are sent to the learning algorithm as human intention (1). Based on gesture features, the learning algorithm selects an action (2). The robot executes the chosen action (3). The subject observes the executed actions of the robot (4). The test person gives an intrinsic feedback on the robot's choice of action in the form of an EEG. The ErrP is evoked, for example, when the action performed by the robot does not match the current human gesture. The output of ErrP decoder (binary classification: ErrP or No ErrP) is sent to the learning algorithm as rewards (5). The learning algorithm updates the policy based on human feedback (6). Dotted lines: Feature vectors of human gesture are written in the log file (r1). Executed actions of the robot are written in the log file (r2) and in the EEG as action markers (h1). EEG signals are continually recorded and saved as EEG data (h2). The outputs of ErrP decoder (rewards) are written in the log file (r3). Payoffs of each gesture-action pair are written in the log file (r4). Details, see sections 1.1 and 2.2.

In our human-robot interaction scenario, the robot learns actions that are best assigned to the *current* human intentions. Our concept of human-robot interaction (HRI) is illustrated in Figure 1. The subject interacts with the robot by selecting a specific gesture that expresses the human intention. The robot observes the current gesture and chooses an action based on the policy from previous trials. The subject observes the chosen action of the robot and evaluates it intrinsically. This intrinsic evaluation is reflected in certain EEG activities, which are a neuronal correlate of the implicit intrinsic evaluation of the correctness of the action of the robot. The robot learns a policy based on human feedback and updates the policy after every other interaction with the subject where further experience is gained. Finally, the robot learns correct mappings between gestures and actions (i.e., correct gesture-action pairs), which is updated in real time by human's online feedback.

The learning algorithm used in our HRI concept is based on a contextual bandit approach (e.g., Li et al., 2010). The contextual bandit approach is well-suited for our HRI scenario, since a robot learns to choose actions which are best assigned with the given context (human's current gestures). The contextual bandit approach is a variant of reinforcement learning, in which only one action is chosen per episode (details, see section 2.2).

Our HRI contains two interfaces between human and robot: (a) gesture interface that encodes human's intents in form of gestures and (b) EEG interface that decodes human's intrinsic feedbacks on robot's actions in form of EEGs. Both interfaces provide inputs to the learning algorithm that triggers actions in the robotic system (robot arm) that are best assigned with the given gestures. Hence, learning performance depends on the quality of inputs that are provided by both interfaces. In our HRI scenario, misinterpretations of human intention (human gesture) and human feedback (human evaluation) affect learning performance. In other words, an incorrect coding of human intention and an incorrect decoding of human feedback has an impact on the learning performance of the robot.

### 1.2. HRI Errors: Gesture Errors and ErrP Misclassifications

In our previous study (Kim et al., 2017) we investigated the effect of ErrP classification performance on robot learning performance, since the results of the ErrP classification are directly used as a reward in the learning algorithm. Thus, we focused on the analysis on ErrP-classification performance. In our HRI scenario, however, the robot receives not only implicit human feedback but also human gestures as explicit input for the interaction. Thus, the robot has two kinds of inputs for interactions with human: (a) human gestures in form of gesture features and (b) human feedback in form of ErrPs, which are neural correlates of human's implicit evaluation on robot's actions. Both types of input can be incorrect in real applications for different reasons.

Gesture errors can be generated when human gestures are not correctly recorded for several reasons. First, hand positions of the subjects are often out of range of sensors (infrared cameras) due to changes of body posture of the subjects. In most cases, the subjects are not aware of such large variances of their own hand positions. Second, in a few cases, we have also a general hardware problem. The gesture recording system called Leap Motion does not accurately enough catch hand gestures due to the limited range of infrared cameras. The accuracy of gesture capture depends on how the subject's hands enter the camera's sensors. Third, some subjects change their gesture patterns during the experiments. For example, at the beginning of the experiment, these subjects made gestures to move the robot to the *right* with their hands open, but in the middle of the experiment they closed their hands before finishing the whole gesture. In this case, an additional gesture feature (e.g., closed hand) was added [1, 0, 0, 1], which is used for the gesture *forward* [0, −1, 0, 1]. Again, the subjects are not aware of their own changes of gesture pattern. An overview of the gesture vector depending on the gesture type is shown in Table 1A. All types of gesture errors provide wrong gesture features to the robot and thus the robot perceives gesture features that are not coherent with gestures that the subjects intended to perform. Therefore, in our data analysis gesture errors are defined as gesture incoherence between human and robot, i.e., incoherence between gestures performed (by humans) and perceived (by robots). Note that maximum values of feature vectors (second column of Table 1A) cannot be reached by actually performed human gestures. We observed individual differences in gesture features within the same gesture type (inter-subject variability) and differences in gesture characteristics between repeatedly executed identical gesture types within the same test subjects (inter-gesture variability).

**Table 1:** **(A)** Four gesture types; **(B)** Errors in human-robot interaction (HRI) and their effects on learning performance.

**Gesture type**		**Feature vector**					**Recorded feature vectors**			
**(A)**										
Left		[−1 0 0 0]					[−0.85 0.11 0.15 0.21]			
Right		[1 0 0 0]					[0.91 0.22 0.32 0.19]			
Upward		[0 1 0 0]					[0.14 −0.84 0.15 0.93]			
Forward		[0 −1 0 1]					[0.11 0.81 0.11 0.23]			
		**Online robot learning**					**Offline analysis**			
		**(a)**	**(b)**	**(c)**	**(d)**	**(e)**	**(f)**	**(g)**	**(h)**	**(i)**
**Case**	**Perception**	**Human**	**Recorded**	**Robot**	**ErrP**	**Rewards**	**Gesture**	**ErrP**	**ErrP**	**Impact on**
		**gesture**	**gesture**	**action**	**detection**		**error**	**error**	**classification**	**learning**
**(B)**										
1	Human	Left	Left	Left	No ErrP	1	No	No	TN	Positive
	robot									
2	Human	Left	Left	Left	ErrP	−0.25	No	Yes	FP	Negative
	robot									
3	Human	Left	Left	Right	No ErrP	1	No	Yes	FN	Negative
	robot									
4	Human	Left	Left	Right	ErrP	−0.25	No	No	TP	Positive
	robot									
5	Human	Left	Right	Left	No ErrP	1	Yes	No	TN	Negative
	robot							Yes	FN	
6	Human	Left	Right	Left	ErrP	−0.25	Yes	Yes	FP	Positive
	robot							No	TP	
7	Human	Left	Right	Right	No ErrP	1	Yes	Yes	FN	Positive
	robot							No	TN	
8	Human	Left	Right	Right	ErrP	−0.25	Yes	No	TP	Negative
	robot							Yes	FP	

*The recorded feature vectors are presented as example. ErrP error stands for ErrP error classification. The positive class stands for faulty action of the robot. Only the cases in which the test subjects perform the gesture to move the robot to the left are described as examples*.

Human feedback (reward) can also be wrong for various reasons. We consider incorrect decoding of human implicit feedback (ErrP) as the most common reason for incorrect human feedback. In general, the accuracy of the trained ErrP decoder is seldom achieved with 100%. Hence, ErrP misclassifications, i.e., both false positives (FP) and false negatives (FN) were counted as erroneous human feedback in our data analysis. Erroneous human feedback can in a few cases also be generated by gesture errors, although there are no ErrP misclassifications (details in section 2.1). Erroneous human feedback can also be caused if the test subjects miss the robot's actions due to lack of attention. In this case, ErrP detections are incorrect and thus erroneous feedbacks are sent to the robot. However, we have found that such errors are indeed rare, since the task (observing the actions of the robot) was actually very simple. This was also shown by the oral feedback of the test persons to our questions, how often they approximately missed the actions of the robot. For this reason, we excluded this type of error from our data analysis.

Both ErrP misclassifications and gesture errors can occur together and influence each other. The interaction of both types of errors can lead to erroneous feedback to the robot, which affects robot learning. The interaction between ErrP misclassifications and gesture errors and their effects on robot learning is reported in detail in section 2.1.

## 2. Methods

### 2.1. Expected Effects of HRI Errors on Learning Performance

**Figure 2:**
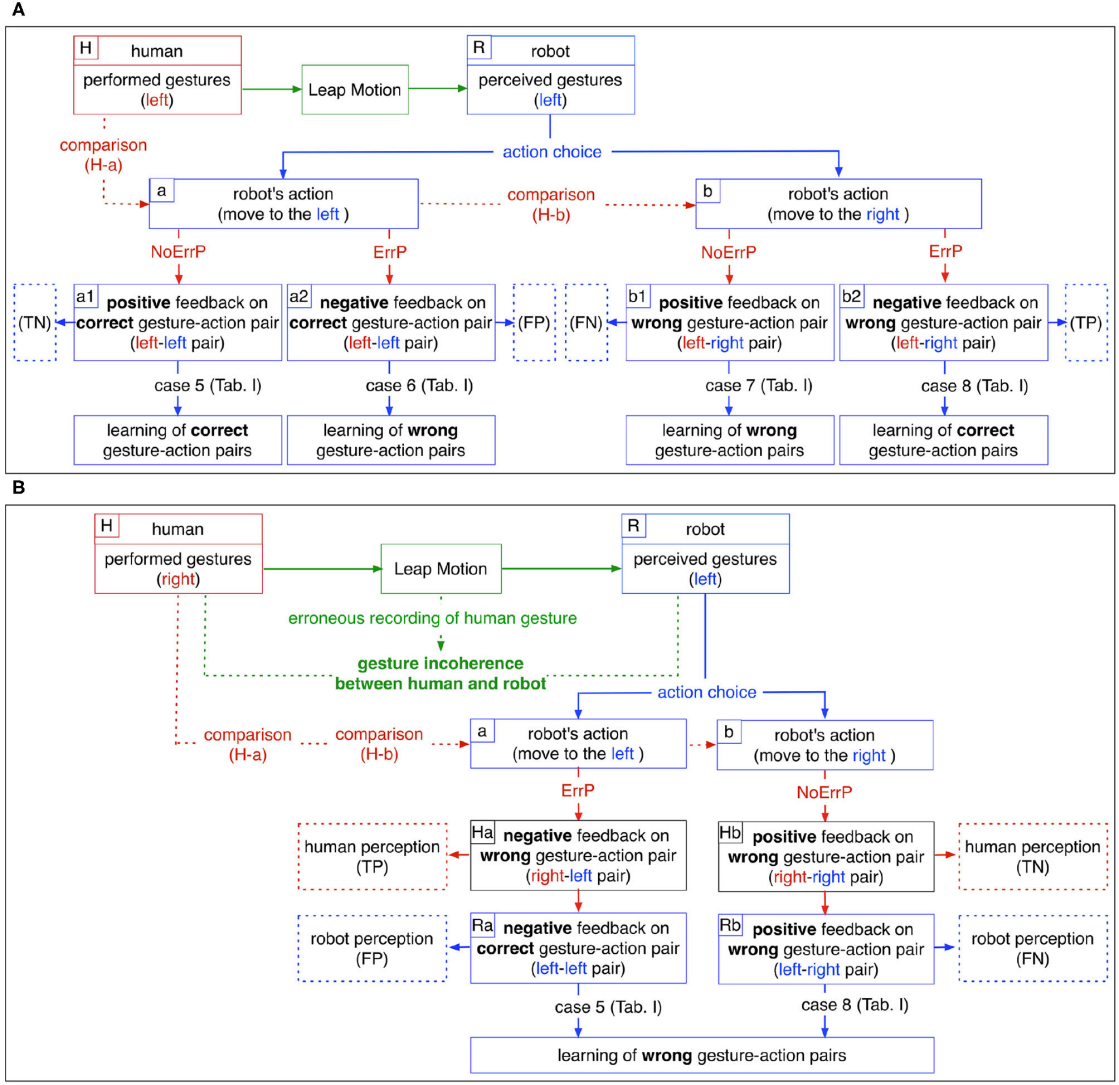
A schematic overview: **(A)** Effects of the ErrP classifications on the learning processes of the robot where there are no gesture errors (no faulty recording of gestures). **(B)** Negative effects of gesture errors on robot learning processes where ErrP recognitions are correct. The cases of ErrP error classifications (case 6 and 7 in Table 1) are not shown. Note that ErrPs are generated based on human perception, while action decisions are based on the perception of the robot (TP, true positive; FP, false positive; TN, true negative; FN, false negative).

Figure 2A shows a schematic overview of the effects of ErrP classifications on the learning process of the robot, where there are no gesture errors (no faulty recording of gestures). ErrPs are used as implicit evaluation of robot's action choice: when ErrPs are detected, negative feedbacks are given to the robot, whereas positive feedbacks are given to the robot when ErrPs are not detected (solid red lines, in Figure 2A). There are two cases for robot learning, when ErrP detections are correct: (a) a positive feedback (No ErrP) is given to a correct gesture-action pair (a1 in Figure 2A) and (b) a negative feedback (ErrP) is given to a wrong gesture-action pair (b2 in Figure 2A). In both cases, the robot learns correct gesture-action pairs (case 5 and 8 in Figure 2A and Table 1B). However, when ErrP detections are wrong, erroneous feedbacks are given to the robot: (a) a negative feedback (ErrP) is given to a correct gesture-action pair (a2 in Figure 2A) and (b) a positive feedback (No ErrP) is given to a wrong gesture action-pair (b1 in Figure 2A). In both cases, the robot learns wrong gesture-action pairs (case 6 and 7 in Figure 2A and Table 1B). Hence, ErrP misclassifications can generate erroneous feedback that negatively affect the learning process in two ways: (a) ErrPs are detected although robot's actions are correct, i.e., false positive (FP) and (b) ErrPs are not detected although robot's actions are wrong, i.e., false negative (FN), where positive class stands for erroneous actions.

Figure 2B shows a schematic overview of the negative effects of gesture errors on the robot's learning performance, where ErrP detections are correct *per se*. Gesture errors can have a direct or indirect effect on the robot's learning performance, but their impact on the learning process is not straightforward, since gesture errors affect ErrP error classifications that further influence the learning process. This means that the effects of gesture errors on the learning process cannot be easily interpreted. When gestures are incorrectly recorded, the performed gestures of human are not coherent with the recorded gestures (green dotted line in Figure 2B). Hence, the robot perceives gesture features that are incoherent with the subject's performed gestures and decides an action based on the perceived gestures. On the other hand, human feedbacks are generated based on the performed gestures of human. In fact, the test subjects always compare their executed gestures (not the recorded gestures) and the robot's action choices (H-a and H-b in Figure 2B). They are not aware of incorrectly recorded gestures, because the test subjects perceive almost no false recordings of their own gestures when interacting with the robot online. Therefore, human feedback to the robot (No ErrP/ErrP) is generated based on the gestures performed by the human, while the robot receives characteristics of the recorded gestures. That means, online-reward generations (ErrP detections) are based on human perception, whereas action choices of the robot are based on robot perception. In the end, erroneous recordings of gestures lead to the generation of incorrect feedback: (a) ErrP with correct gesture-action pairs (Ra in Figure 2B) and (b) No ErrP on an incorrect gesture-action pair (Rb in Figure 2B), although the ErrP detections are correct in themselves, i.e., there are no ErrP misclassifications (Ha and Hb in Figure 2B).

For schematic overviews, we visualized the effect of ErrP classifications (rewards) without gesture errors (Figure 2A) or the effect of gesture errors without ErrP misclassifications (Figure 2B). However, ErrP misclassifications and gesture errors can occur together and interact.

Table 1B shows all theoretically possible cases of input errors (gesture errors/ErrP misclassifications) and their combinations in our HRI scenario. In Table 1B only the cases are exemplarily described in which the subjects perform the gesture to move the robot to *left*.

When there are no gesture errors (case 1, 2, 3, 4 in Table 1B), ErrP-classification performances are same for both human perception and robot perception (Table 1B-h). When gesture errors are observed (case 5, 6, 7, 8 in Table 1B), ErrP-classification performances are different between human perception and robot perception (Table 1B-h). Gesture errors have a negative effect on the robot's learning process if they occur without ErrP error classifications (case 5 and 8 in Table 1B), because the robot learns gesture-action pairs based on the recorded gestures and not on the executed human gestures and receives erroneous feedback from the test persons (case 5: No ErrPs on *right*-*left* pairs; case 8: ErrPs on *right*-*right* pairs). However, when gesture errors and ErrP misclassifications occur together, learning performances of the robot are positively affected, since gesture errors cancel out ErrP misclassifications (case 6 and 7 in Table 1B) and the robot receives correct feedbacks from the subjects (case 6: ErrP on *right*-*left* pairs; case 7: No ErrP on *right*-*right* pairs).

In summary, misinterpretations of human intention (gesture errors) and human feedback (ErrP error classifications) can separately influence the learning process as follows: Learning process can be negatively affected by (a) ErrP misclassifications without gesture errors (case 2 and 3 in Table 1B) or (b) gesture errors without ErrP misclassifications (case 5 and 8 in Table 1B). However, in a few cases, there is an interaction between gesture errors and ErrP misclassifications, which positively affects the learning process, since gesture errors cancel out ErrP misclassifications (case 6 and 7 in Table 1B). Finally, the absence of both error types (correct gesture recordings and correct ErrP detections) has a positive impact on the learning process (case 1 and 4 in Table 1B).

### 2.2. Learning Algorithm

In our HRI scenario, a robot learns to choose actions which are best assigned with the given context (human's current gestures), in which robot's actions have single-state episodes and the context is independent of each other. Thus, the contextual bandit approach is well-suited for our HRI scenario. Among state-of-the art contextual bandits approaches, we chose LinUCB (Li et al., 2010) as learning algorithm (see Algorithm 1). In principle, LinTS (Agrawal and Goyal, 2013) is also suitable for our HRI scenario. Although both algorithms are interchangeable, empirical evaluation of both algorithms led to different learning performances depending on application scenarios (Chapelle and Li, 2011). Further, other state-of-art algorithms regarding multi-arm bandits can also be implemented for contextual bandits settings (Cortes, 2018). However, LinUCB (Li et al., 2010) is a popular approach that has been evaluated in numerous scenarios and proved as a fast and effective approach in contextual bandit settings [e.g., HybridLinUCB (Li et al., 2010), GOB.Lin (Cesa-Bianchi et al., 2013), CLUB (Gentile et al., 2014), CoLin (Wu et al., 2016)].

**Algorithm 1:** LinUCB (Li et al., 2010)

0: Inputs: α ∈ ℝ_+_
1: **for** *t* = 1, 2, 3, …, *T* do
2: Observe features of all arms *a* ∈ A_*t*_: *x*_*t,a*_ ∈ ℝ^*d*^
3: **for all** *a* ∈ A_*t*_ **do**
4: **if** *a* is new **then**
5: *A*_*a*_ ← *I*_*d*_ (d-dimensional identity matrix)
6: *b*_*a*_ ← 0_*d*×1_ (d-dimensional zero vector)
7: **end if**
8: ${\hat{\theta }}_{a}$ ← $A_{a}^{-1}$ *b*_*a*_
8: *P*_*t,a*_ ← ${\hat{\theta }}_{a}^{T}$ *x*_*t,a*_ + α $\sqrt{x_{t,a}^{T}A_{a}^{-1}x_{t,a}}$
10: **end for**
11: Choose arm *a*_*t*_ = *arg* ${max}_{a\in A_{\text{t}}}$ *P*_*t,a*_ with ties broken arbitrarily and observe a real valued payoff *r*_*t*_
12: *A*_*a*_*t*__ ← *A*_*a*_*t*__ + *x*_*t*,*a*_*t*__ $x_{t,a_{t}}^{T}$
13: *b*_*a*_*t*__ ← *b*_*a*_*t*__ + *r*_*t*_ *x*_*t*,*a*_*t*__
14: **end for**

**Figure 3:**
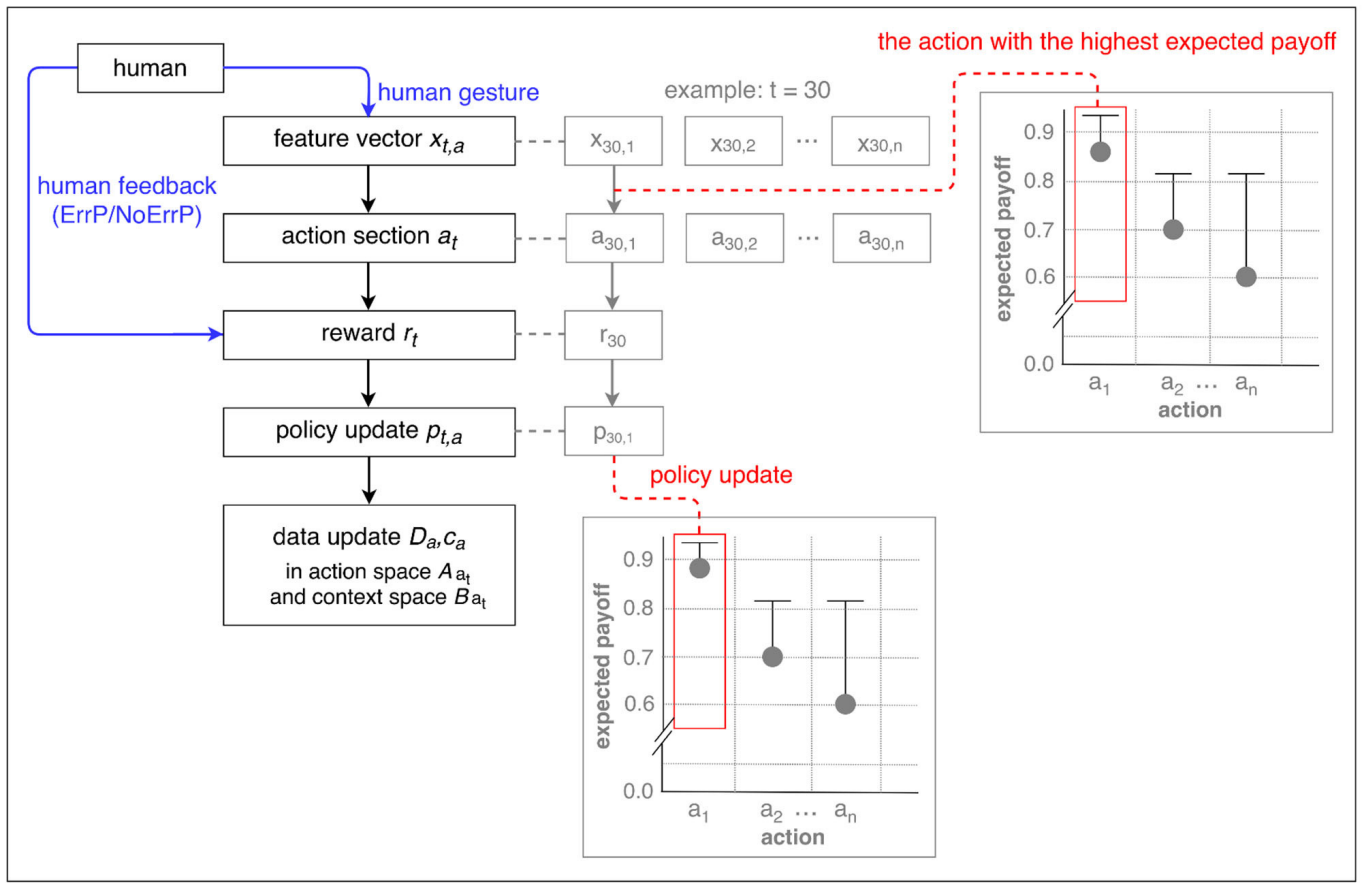
A schematic visualization of LinUCB (Li et al., 2010). Action selection and policy update were depicted in the given context *x*_1_ for the 30th trial (episode) as an example. In accordance with our HRI scenario, the subjects performed the *left gesture* (*x*_30,1_) among other gesture types (*x*_30,2_, *x*_30,3_, *x*_30,4_) in the current episode (in the 30th episode). In this example, a correct action *x*_1_ (left action of the robot) is chosen in the given context *x*_1_ (left gesture). The policy [i.e., the expected payoff that is equivalent to the upper confidence bound (UCB)] is updated for the chosen action, i.e., *x*_1_-*a*_1_ pair.

Contextual bandits (Langford and Zhang, 2008) have single-state episodes, since they obtain only one immediate reward per episode. This is similar to *k*-armed bandits (Auer et al., 2002) that is the simplest form of reinforcement learning. However, contextual bandits use the information about the state of the environment (cf. *k*-armed bandits) and thus make decision dependent on the state of the environment (context). That means, the policy of context (state)-action pair is updated per episode (trial) and the context is independent of each other. Accordingly, the context is different for each episode (trial). For example, in our HRI scenario, the subject performs different types of gesture (left, right, forward, upward) for each episode, e.g., *left gesture* (*x*_1,1_) for the first episode, *right gesture* (*x*_2,2_) for the second episode, *left gesture* (*x*_3,1_) for the third episode, *forward gesture* (*x*_4,3_) for the fourth episode, etc. Figure 3 shows a schematic visualization of LinUCB (Li et al., 2010) in a given context in a specific episode as an example.

In LinUCB (Li et al., 2010), it is assumed that the predicted payoff (the expected payoff) of an arm *a* is linear in its *d*-dimensional feature *x*_*t,a*_ with some unknown coefficient vector $\theta_{a}^{*}$: $E\left[r_{t,a}|x_{t,a}\right]=x_{t,a}^{T}\theta_{a}^{*}$. Note that the model is called disjoint, since the parameters are not shared among different arms. Ridge regression is applied to the training data (*D*_*a*_, *c*_*a*_) in order to estimate the coefficients $\theta_{a}^{*}$ (details, see below). The algorithm observes feature vector *x*_*t*_ and selects an action *a*_*t*_ based on *the predicted payoffs* of all actions. After action selection, the algorithm receives *the current payoff* *r*_*t*,*a*_*t*__ and updates the policy with the new observation (*x*_*t*,*a*_*t*__, *a*_*t*_, *r*_*t*,*a*_*t*__). The step-by-step description follows below (see Algorithm 1).

The exploration parameter α is determined before the learning was used as input (line 0). For each time, e.g., for each trial (line 1), the algorithm observes all features (line 2). When the action has not been observed before (line 4), one *d* × *d* identity matrix (*I*_*d*_) and one zero vector of length *d* (0_*d*×1_) are instantiated (line 5, line 6), where *d* is the number of features. The coefficient ${\hat{\theta }}_{a}$ is estimated by applying ridge regression to the training data (*D*_*a*_, *c*_*a*_), where *D*_*a*_ is a *m* × *d* design matrix and *c*_*a*_ is the vector of length *m* (where *m* is the number of observations): ${\hat{\theta }}_{a}$ = ${\left(D_{a}^{T}D_{a}+I_{d}\right)}^{-1}$ $D_{a}^{T}c_{a}$. In the Algorithm 1, $D_{a}^{T}$ *D*_*a*_ + *I*_*d*_ is rewritten as *A*_*a*_ and $D_{a}^{T}c_{a}$ is rewritten as *b*_*a*_ (line 8). Accordingly, ${\hat{\theta }}_{a}$ can be rewritten as $A_{a}^{-1}$ *b*_*a*_. Payoffs *P*_*t,a*_ are estimated as the sum of ridge regression for the current feature *x*_*t,a*_ (i.e., the expected payoff: ${\hat{\theta }}_{a}$ *x*_*t,a*_) and the standard deviation of the expected payoff ($\sqrt{x_{t,a}^{T}A_{a}^{-1}x_{t,a}}$), where the standard deviation is multiplied by the parameter α that determines the degree of exploration (line 9). The algorithm chooses the action with the highest expected payoff (*arg* ${max}_{a\in A_{\text{t}}}$ *P*_*t,a*_) and observes the received *current* payoff *r*_*t*_ on the chosen action (line 11). Finally, the training data (*D*_*a*_, *c*_*a*_) is updated in action space *A*_*a*_*t*__ and context space *b*_*a*_*t*__ (line 12 and line 13), which is fitted by applying ridge regression to estimate ${\hat{\theta }}_{a}$ for the next trial. Therefore, the expected payoff is linear in its *d*-dimensional feature *x*_*t,a*_ with some unknown coefficient vector $\theta_{a}^{*}$: $E\left[r_{t,a}|x_{t,a}\right]=x_{t,a}^{T}\theta_{a}^{*}$. Payoffs *p*_*t,a*_ are affected by two parameters: the expected payoff (exploitation) and the standard deviation of the expected payoff (exploration). The optimum of action strategy is obtained by balancing exploration and exploitation.

In our HRI scenario, the algorithm learns to select robot's actions *a*_*t*_ that are best assigned with the current context *x*_*t*_, i.e., the current human intention in form of gesture feature recorded by the Leap Motion. The current payoff, i.e., the immediate reward is the ErrP-classification output (ErrP or No ErrP), which is given to the action chosen by the LinUCB algorithm, i.e., the executed action of the robot. As mentioned earlier, action selection was made conditional on human gesture (left, right, forward, upward). We call actions together with gesture features “gesture-action pairs” (i.e., context-action pairs). The LinUCB algorithm learns a correct mapping between human gesture features and actions of the robot, i.e., a correct gesture-action pair. In fact, the robot should learn which action is correctly executed. Hence, our HRI scenario is designed that the predictions of correct mappings (No ErrP) are highly rewarded [1] than the predictions of wrong mappings (ErrP) that are minimally punished [−0.25]. To this end, we used two windows for the same action in online ErrP detection and the predictions of correct mappings (No ErrP) were sent to the learning algorithm, only when No ErrP was predicted from both time windows (Table 2). As a result, the rewards for predicted correct mapping (TN, FN) were weighted more strongly than predicted wrong mapping (FP, TN). Note that the reward values of [−0.25, 1] were empirically determined. Further, the exploration parameter α was also empirically determined [α = 1].

**Table 2:** Use of ErrP detection as a reward in the learning algorithm.

(A) Actual label	Correct	Correct			Wrong	Wrong		
(B1) Prediction (1st window)	No ErrP	No ErrP	ErrP	ErrP	No ErrP	No ErrP	ErrP	ErrP
(B2) Prediction (2nd window)	No ErrP	ErrP	No ErrP	ErrP	No ErrP	ErrP	No ErrP	ErrP
(C) Predicted label	No ErrP (correct)	ErrP (wrong)			No ErrP (correct)	ErrP (wrong)		
(D) Rewards	1	−0.25			1	−0.25		
(E) ErrP evaluation	TN	FP			FN	TP		

*Actual labels are obtained by comparing between gesture labels and action labels (gesture-action pairs). The outputs of ErrP decoder, i.e., predictions (B1 and B2) are obtained by two windows with the same robot actions (same gesture-action pair). A decision was made from two windows, and this is used as predicted labels (C) for the confusion matrix. Rewards (D) are sent to the learning algorithm (online learning). The evaluation of the ErrP classifications (ErrP detections) is based on the confusion matrix*.

One of the key elements of our approach is to adapt the previous learned policy when changing the current human intention (i.e., when changing the semantics of gestures). Thus, human gesture was not predefined, i.e., no initial semantics of gestures was given to the robot. Rather, the robot learned the current meaning of human gesture, which can be changed online. That means, there were no fixed labels (no fixed semantics of gestures) to train a model. For this reason, we did not train a classifier to distinguish different types of *predefined* gestures. Instead, the robot received gesture feature vectors recorded by the Leap Motion instead of classified gestures. Accordingly, no *classified* gestures were sent to the robot. The chosen algorithm called LinUCB enables to learn gesture-action pairs without prior knowledge of gesture meaning.

In fact, we observed a variation of gesture feature vectors between trials within the same subject (details, see section 1.2), but this did not prevent robust learning of gesture-action pairs. Learning remains robust due to the updates of context space per trial: The current context, i.e., gesture feature vector (*x*_*t,a*_) was added to the context space (*b*_*a,t*_) together with the corresponding current payoff (*r*_*t,a*_) for each trial. This update of the context space allows for robust learning despite of variations of gesture feature vectors between trials (e.g., [−0.9, 0.15, 0.29, 0.37], [−0.8, 0.27, 0.41, 0.05], [−0.95, 0.29, 0.11, 0.88], etc.) for the *left* gesture type (default value [−1, 0, 0, 0]). In this way, gesture feature vectors were adapted per trial within a subject.

The main scope of this study was to analyze erroneous inputs and their impacts on learning performance. The data analysis was based on the log files that were generated for each online experiment. Note that learning was completed for each online experiment (i.e., each online dataset). The following outputs were logged online per trial within an online experiment:

- Updates of action space *A*_*a*_ and context space *b*_*a*_ (line 5 and 6 in Algorithm 1)
- Gesture feature vector for the current trial *x*_*t,a*_ (line 1 in Algorithm 1, Figure 1-r1)
- The action with the highest expected payoff for the current trial *a*_*t*_ (line 11 in Algorithm 1, Figure 1-r2)
- The current payoff, i.e., the immediate reward *r*_*t*_ for the current trial (line 11 in Algorithm 1, Figure 1-r3)
- The expected payoffs *P*_*t,a*_ (line 9 in Algorithm 1, Figure 1-r4)

Gesture feature vectors were logged automatically while online learning (Figure 1-r1). That means, gesture feature vectors recorded by Leap Motion was logged online. However, human gestures, i.e., gestures performed by human could not be logged online. Thus, we filmed human gestures and robot's actions during online experiments. After experiments, we investigated which gesture feature vectors were perceived by the robot. To this end, we reconstructed gestures per trial based on the logged gesture feature vectors in the log file. Such reconstruction was done only for offline data analysis. We used the following decision criteria for reconstruction of gestures: (a) *m* = 1, if *m* > 0.5 (b) *m* = −1, if *m* < −0.5 (c) *m* = 0, if −0.5 < *m* < 0.5, where *m* is each component of vector. The gesture feature vector consists of four components (details, see section 2.3.4). In this way, we obtained filmed gestures and reconstructed gestures. Finally, gesture errors were estimated by comparing filmed human gestures (e.g., *left* gesture) and the reconstructed gesture based on recorded gesture vectors [e.g., −0.8, 0.1, 0.2, 0.1]. Further, filmed gestures were used to determine the correctness of gesture-action pairs and to find a true label to generate a confusion matrix for human's perspective, whereas the reconstructed gestures were used as a true label to generate a confusion matrix for robot's perspective (details, see section 2.4).

### 2.3. Scenario and Dataset

We used the data that was recorded in the previous study for investigation on flexible adaptation of learning strategy using EEG-based reinforcement signals in real-world robotic applications (Kim et al., 2020). In the previous study (Kim et al., 2020), data was recorded from eight subjects (2 females, 6 males, age: 27.5 ± 6.61, right-handed, normal or corrected-to normal vision). The experiments were carried out in accordance with the approved guidelines. Experimental protocols were approved by the ethics committee of the University of Bremen. Written informed consent was obtained from all participants that volunteered to perform the experiments.

In our HRI scenario (Kim et al., 2017), the subjects perform gestures (left, right, forwards) and observe the robot's actions as response to the human gestures (Details, see section 1.1 and Figure 1). In the extend HRI scenario (Kim et al., 2020), the subjects add a new gesture (upwards) after about 30 trials, while the robot still learns the mapping between human gestures and its own actions. That means, the subjects determine the meaning of the gesture (human intent) and select one of gestures. The robots learns to select an action that is best assigned to the current human intents (current gesture) based on human implicit feedback in form of EEG. The goal of the previous study was to investigate whether the robot can flexibly adapt the learning strategy in real time, when the user changes the *current* intentions (in form of EEG). For example, the subjects changed their control strategy e.g., by adding a new context (gesture) to the previous used gestures. Our results showed that the robot could adapt the previously learned policy depending on online changes of the user's intention (Kim et al., 2020). This investigation was validated under two learning conditions: (a) learning algorithm was trained with a few samples (1 or 2 gesture-action pairs) before online learning (pretraining) and (b) learning algorithm was not trained before online learning (no-pretraining).

#### 2.3.1. Scenario Description

In the previous study (Kim et al., 2020), we collected data in two different scenarios: (a) observation scenario and (b) interaction scenario. In the *observation* scenario, the subjects observed the robot's action. Here, the subjects were not required to interact with the robot, e.g., by performing gestures, since human gestures and robot's action choice were already preprogrammed. A hand gesture was displayed to the subjects as a word (left, right, forward, or upward) on the monitor, which is located on the left side of the robot. Then, a feature vector of the displayed gesture (Table 1A, second column) was sent to the pseudo-learning algorithm, where action selections were preprogrammed. Gesture-action pairs are preprogrammed with the class ratio of 8:1 (correct/wrong actions). The observation scenario was designed to train a ErrP classifier in order to detect ErrPs online in the interaction scenario. In the observation scenario, the subjects did not perform gestures and the robot did not learn any action selection strategy. In this way, we could reduce the recording time for training data for ErrP decoder. We trained a classifier for each subject to distinguish ErrP and No ErrP, which was later used to detect ErrPs in the *interaction* scenario. Such classifier transfer was successfully evaluated in our previous studies (Kim and Kirchner, 2013, 2016; Kim et al., 2017).

In the *interaction* scenario, the subjects performed one of four gesture types (left, right, forward, and upward). As mentioned before, we used the Leap Motion to record human gestures. Gesture feature vectors recorded by Leap Motion were sent to the LinUCB algorithm. Then, the algorithm selected an action and sent this action selection to the robot. The subject observed the action choice of the robot and at the same time the implicit evaluation of the chosen action of the robot was measured by using the EEG and the so called ErrP was detected online per action choice.

Implicit human evaluations (ErrP/No ErrP) were sent to the LinUCB algorithm as rewards.

#### 2.3.2. Datasets for Training of ErrP Decoder (Observation Scenario)

For training a classifier (ErrP decoder), we recorded data in the *observation* scenario, in which the subjects observe the robot's actions without performing a gesture to reduce the recording time of EEG data. The subjects were instructed to observe the gesture that was displayed as a word (left, right, forward, or upward) on the monitor. After the display of the gesture disappeared on the monitor, the robot started to move the arm. The subjects were instructed to observe the actions of the robot. Six datasets were recorded from each subject. Each dataset consists of 80 correct actions of the robot and 10 wrong actions of the robot (90 instances in total). Gesture-action pairs are preprogrammed with the class ratio of 8:1 (correct/wrong actions). We had a uniform number of training dataset, i.e., all participants had the same number of training dataset (six datasets).

#### 2.3.3. Online Datasets During Robot Learning (Interaction Scenario)

In the online application (i.e., online EEG-based RL learning), the subjects performed gestures to communicate with the robot. To this end, we used the *interaction* scenario. The subjects were instructed to freely perform one of three gestures (left, right, forward, see Table 1B) and add the fourth gesture (upward, see Table 1B), when they heard a short tone that was given to the subjects after 30 trials. Before the start of the online experiments in the interaction scenario, all subjects had a short practice set to train the correct use of Leap Motion.

The robot chooses an action as response of the current human intention (human gesture) and receives an immediate reward in form of ErrP-classification output [ErrP/No ErrP]. The robot updates the policy based on human feedback (details, see section 2.2).

##### 2.3.3.1. Learning condition

Two learning conditions were investigated in online learning: warm-start learning (pre-training) and cold-start learning (no pre-training). In warm-start learning, a few trials (# of trial *n* < 4) were pre-trained, i.e., a few gesture-action pairs were trained with the perfect human feedback (i.e., the perfect ErrP-classifications). That means, the perfect human feedback was given to the action choice of the robot that was preprogrammed. Hence, we expected less erroneous actions of the robot (i.e., less mapping errors) in the beginning of learning phase for warm-start learning compared to cold-start learning. Note that the three kinds of gestures (left, right, forward) were pre-trained, but not the fourth gesture (upward) that was added during learning process online. In cold-start learning, we did not pre-train any gesture-action pairs. For all subjects, we started with the warm-start learning condition before the cold-start learning conditions to prevent the frustration of subjects, which can be caused by a large number of erroneous actions of the robot in cold-start learning. We did not alternate both learning conditions within subjects.

##### 2.3.3.2. Number of trials in both learning conditions

In warm-start learning, we used the same number of trials for all subjects (90 trials). In cold-start learning, we used the same number of trials for all subjects (90 trials) except for one subject (60 trials, 90 trials, 120 trials for each online dataset). In fact, we investigated a different number of trials to find the appropriate number of trials. We aimed to find when the learning curve is stabilized (no mapping errors). To this end, we started with 120 trials and reduced the number of trials (90 trials, 60 trials). We did this evaluation on the first subject. In total, three datasets with 120 trials were recorded from the first subject. We reduced gradually the time to give a short tone for adding a new gesture. In the first dataset, the short tone was given to the subject after 60 trials (**Figure 7**). In the second dataset, the short tone was given to the subject after 50 trials (**Figure 5**). In the third dataset, the short tone was given to the subject after 40 trials. Finally, we decided to give a short tone for adding a new gesture after 30 trials. Based on this analysis, 60 trials were already enough for convergence in this subject. However, we are aware of subject variability in ErrP-classification performance and that for some subjects more trials might be needed. Moreover, we also did not intend to record on different days due to changes of electrode positions. Actually, the duration of the dataset with 120 trials was 32 min. This would have been too long for one session in total. Hence, we determined 90 trials for online dataset in both learning conditions. That means, there was no difference in the number of trials between warm-start learning and cold-start learning. Note that the first two datasets with 120 trials were excluded for statistical analysis (inference statistics). However, we included them for descriptive analysis and visualization for three reasons: (a) descriptive visualization of the learning curve in different number of trials (90 trials vs. 120 trials; **Figures 5A,B** vs. **Figures 5C,D**), (b) descriptive visualization of gesture errors (i.e., incoherence between human's perspective and robot's perspective, see **Figure 7**, **Table 5**), and (c) descriptive visualization of a few number of gesture errors (**Figure 5**, **Table 6**) and a large number of gesture errors (**Figure 7**, **Table 5**).

##### 2.3.3.3. Number of online datasets in both learning conditions

In warm-start learning, we recorded three online datasets for four subjects and two online datasets for four subjects. In total, we recorded 20 datasets in warm-start learning. In cold-start learning, two online datasets were recorded for five subjects and three online sets were recorded for two subjects. For one subject, we recorded only one online dataset. This participant was very tired after recording the online dataset. Thus, we did not record further online datasets, since this participant could not concentrate on the task. In total, we recorded 17 datasets in cold-start learning. It is worth noting that the number of online datasets has no impact on the learning performance of the robot or ErrP-classification performance, since the learning process is completed within an online dataset (online experiment) and thus the learning of online datasets is independent of each other. It is thus enough to record only one online dataset (online experiment) per subject. However, we recorded more than one online dataset to obtain more data for this evaluation, in case that a participant allowed us to record more than one online dataset. The number of online datasets for each subject and each learning condition was reported in Supplementary Table 1. As shown in Supplementary Table 1, there was no high difference between learning conditions within subjects. Note that the different number of datasets between learning conditions were taken into consideration in inference statistics.

#### 2.3.4. EEG Recording, Gesture Recording, and Robot Arm

For both scenarios (interaction/observation), EEG were continuously recorded using the with 64-channel actiCap system (Brain Products GmbH, Munich, Germany), sampled at 5 kHz, amplified by two 32 channel Brain Amp DC amplifiers (Brain Products GmbH, Munich, Germany), and filtered with a low cut-off of 0.1 Hz and high cut-off of 1 kHz. Impedance was kept below 5 kΩ. The EEG channels were placed according to an extended standard 10–20 system.

For recording of human gesture, we used the Leap Motion system (Leap Motion Inc., San Francisco, USA). The Leap Motions uses a stereo image generated by using two monochromatic infrared cameras. The positions of hand and finger bones can be detected in *x*, *y*, and *z* coordinates relative to the sensor. We used the *x*, *y*, *z* components of the palm normal vector and a value from 0 to 1, which describes how far the hand is opened or closed. (flat hand [0], fist [1]). We recorded ten samples with the length of 100 ms per gesture and averaged them. Gesture feature vectors were used as inputs (human intention) for the LinUCB algorithm. Four types of gestures were used in the experiments: left, right, forward, and upward (see Table 1A). Gesture features recorded by LeapMotion were logged online (Figure 1-r1, details, see section 2.2). Additionally, we filmed online experiments to record gestures performed by human. In this way we received both gestures performed by humans (*gestures*) and gestures perceived by the robot (*gestures*).

The LinUCB algorithm selects actions, which were sent to a six degree of freedom (6-DOF) robotic arm called COMPI (Bargsten and Ferandez, 2015), which was developed at our institute (RIC, DFKI, Germany). We implemented six predefined actions (left, right, forward, upward, and back to start) in joint space, which were triggered from the LinUCB algorithm.

### 2.4. Data Analysis

For analysis of EEG data, we used a Python-based framework for preprocessing and classification (Krell et al., 2013). The EEG signal was segmented into epochs from −0.1 to 1 s after the start of the robot's action for each action type (correct/wrong trial). All epochs were normalized to zero mean for each channel, decimated to 50 Hz, and band pass filtered (0.5–10 Hz). We used the xDAWN spatial filter (Rivet et al., 2009) for feature extraction and 8 pseudo channels were obtained after spatial filtering. Two windows were used for the same robot's action and thus features were extracted from two windows (8 pseudo channels): [−0.1–0.6 s, 0–0.7 s] and normalized over all trials. A total of 280 features (8 pseudo channels × 35 data points = 280 for each sliding window) were used to train a classifier. A linear support vector machine (SVM) (Chang and Lin, 2011) was used for classification.

In this study, we performed two main analyses: (a) learning performance of the robot (mapping errors) and (b) ErrP-classification performance (rewards for learning algorithm). For evaluation of learning performance of the robot, we evaluate the correctness of gesture-action pairs by comparing between human gestures and robot's actions. For evaluation of ErrP-classification performance, we generated a confusion matrix based on the outputs of ErrP decoder (predicted label) with the correctness of gesture-action pairs (actual label).

For example, when gestures performed by human and actions of the robot are identical (e.g., gesture: *left*; action: *left*), robot's actions are correct, i.e., gesture-action pairs (*left-left* pairs) are correct. When ErrPs are detected on *correct* gesture-action pairs (e.g., *left-left* pairs), predictions of the ErrP decoder are wrong (FP). Otherwise, predictions of the ErrP decoder are correct (TP). In contrast, if ErrPs are not detected on *wrong* gesture-action pairs (e.g., *left-right* pairs), ErrP classifications are wrong (FN). Otherwise, predictions of the ErrP decoder are correct (TN). Note that the positive class stands for a wrong action of the robot.

Hence, evaluations of robot's performance and ErrP-classification performance are straightforward, when gestures performed by human and gestures recorded by LeapMotion are identical (i.e., there occur no gesture errors). In this case, the logs of learning process (Figure 1-dotted lines) are enough for evaluation of robot's learning performance and ErrP-classification performance. For example, we can evaluate the correctness of robot's actions by comparing gesture features (Figure 1-r1) with executed actions (Figure 1-r2). We can also evaluate ErrP-classification performance by comparing the output of ErrP decoder (Figure 1-r3) with gesture (Figure 1-r1)-action (Figure 1-r2) pair.

However, there were incoherences between gestures perceived by the robot (recorded gestures) and gestures performed by human, which result in two different perspectives (Table 1B and Figure 2B). Such incoherences between human perception and robot perception can affect the robot's learning performance, since ErrPs are elicited by (*performed*) gesture-action pairs, whereas the learning algorithm updates the current strategy based on (*perceived*) gesture-action pairs (details, see section 1.2). For this reason, data was analyzed in both perspectives (human/robot). For human's perspective, the correctness of robot's actions was calculated by comparing *filmed gestures* with robot's actions, where we filmed human's action while performing gestures. For robot's perspective, the correctness of robot's actions was calculated by comparing *reconstructed gestures* with robot's actions, where we reconstructed gestures based on gesture features recorded by Leap Motion. Therefore the ErrP classification performance was also different between the human and the robot perspective, because the correctness of the robot actions (actual marking) was different between both perspectives (Table 1B).

Finally, four steps of data analysis were performed. First, we evaluated learning performance of the robot (mapping errors) and learning progress of the robot in the whole learning phase. Further, we evaluated the changes of learning progress after changing the current human intention. To this end, we divided the whole learning phase in three learning phases according to the time point of when a new gesture (changes of human intents) was added. In this way, we determined three learning phases: (a) beginning phase (start-1/3), (b) phase after adding a new gesture (1/3-2/3), and (c) final phase (2/3-end). Second, we evaluated ErrP-classification performance in the whole learning progress. Third we analyzed the effect of ErrP-classification performance on learning performance by comparing the pattern of learning progress in mapping errors and the pattern of learning progress in ErrP-classification performance. Fourth, we computed gesture errors by calculating incongruence between robot's perception and humans' perception to analyze the effect of gesture errors on learning performance of the robot. Finally, we analyzed the interaction effect of gesture errors and ErrP misclassifications on learning performance of the robot. All analyses were performed under both learning conditions (warm-start learning and cold-start learning) as well as under both perspectives (human's perspective and robot's perspective).

### 2.5. Statistical Analysis

We investigated the effect of interaction errors (ErrP misclassification, gesture errors) on robot's learning performance (mapping errors) in both learning conditions (cold-start learning, warm-start learning), both perspectives (human's perspective, robot's perspective), and three learning phases (beginning phase, phase after adding a new gesture, final phase). To this end, three factors were designed in statistics: learning condition (two levels: cold-start learning, warm-start learning), perspective (two levels: human's perspective, robot's perspective), and learning phase (three levels: beginning phase, phase after adding a new gesture, final phase).

**Figure 4:**
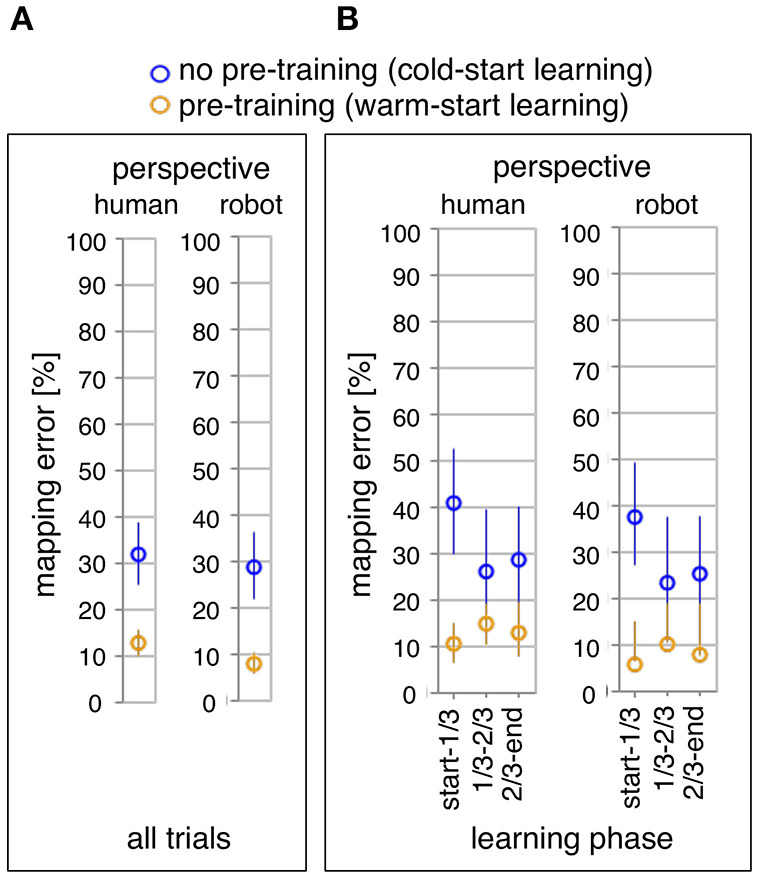
Online learning performance of the robot: the average of mapping errors across all datasets for the whole learning phase **(A)** and the three learning phases **(B)**. Mapping errors are presented for both perspectives: the human and the robot perspective. For each perspective, both learning conditions are compared: pre-training (yellow) vs. no pre-training (blue).

For statistical analysis, we performed six investigations to find out (a) effects of learning condition, learning phase, and perspective on learning performance of the robot (mapping errors), (b) effects of learning condition, learning phase, and perspective on ErrP misclassifications (FN ∪ FP), (c) effects of learning condition, learning phase, and perspective on TP, (d) effects of learning condition, learning phase, and perspective on FN, (e) effects of learning condition, learning phase, and perspective on TN, and (f) effects of learning condition, learning phase, and perspective on FP (see Figures 4B, **6B,D,F,H**; for a descriptive analysis of both robot's learning performance and ErrP-classification performance, see Table 3).

**Table 3:** The mean ErrP-classification performance across all subjects and the standard error of the mean for both learning conditions and both perspectives.

	**Human's perspective**		**Robot's perspective**	
	**Cold-start learning**	**Warm-start learning**	**Cold-start learning**	**Warm-start learning**
Mapping error	31.75 ± 5.79	12.61 ± 1.85	28.26 ± 6.11	7.84 ± 1.35
Gesture error	5.70 ± 1.65	5.88 ± 1.24	5.70 ± 1.65	5.88 ± 1.24
ErrP misclassifications	23.12 ± 4.56	12.81 ± 1.66	23.89 ± 4.54	13.13 ± 1.80
FN	15.33 ± 4.72	3.96 ± 0.73	14.14 ± 4.85	1.73 ± 0.41
FP	7.79 ± 1.82	8.85 ± 1.36	9.74 ± 2.06	11.40 ± 1.60
TP	16.42 ± 2.26	8.66 ± 1.43	14.46 ± 2.22	6.11 ± 1.10
TN	60.45 ± 5.76	78.53 ± 2.75	61.65 ± 5.92	80.76 ± 2.63
FNR (1-TPR)	37.26 ± 5.34	28.80 ± 4.42	34.90 ± 5.71	19.20 ± 3.93
FPR (1-TNR)	13.57 ± 3.28	10.52 ± 1.67	15.45 ± 3.23	12.72 ± 1.88

*FNR = FN/(FN+TP), FPR = FP/(FP+TN), ErrP misclassification = FN ∪ FP*.

To this end, a three-way repeated measures ANOVA was performed with learning condition (2 levels: warm-start learning, cold-start learning) as between-subjects factor and perspective (2 levels: human's perspective, robot's perspective) and learning phase (3 levels: beginning phase, phase after adding a new gesture, final phase) as within-subjects factors. Note that the sample size was unequal for learning condition, since one subject performed only one online experiment (online dataset) in the cold-start learning condition. For this reason, the independent variable *learning condition* was considered as between-subjects factor in the three-way repeated measures ANOVA. Dependent variables were robot's learning performance (mapping errors), ErrP-classification performance, e.g., misclassifications (FN ∪ FP), FN, FP, TN, and FP. For each dependent variable, we separately performed the three-way repeated measures ANOVA. Greenhouse Geisser correction was applied if necessary. Three *post-hoc* analyses were performed, i.e., pairwise comparisons were performed at each factor to compare (1) both learning conditions for each perspective (human's perspective vs. robot's perspective), (2) both perspectives for each learning condition (warm-start learning vs. cold-start learning), and (3) three learning phases for each learning condition and each perspective (beginning phase vs. phase after adding a new gesture vs. final phase). Bonferroni correction was performed for pairwise comparisons.

**Figure 5:**
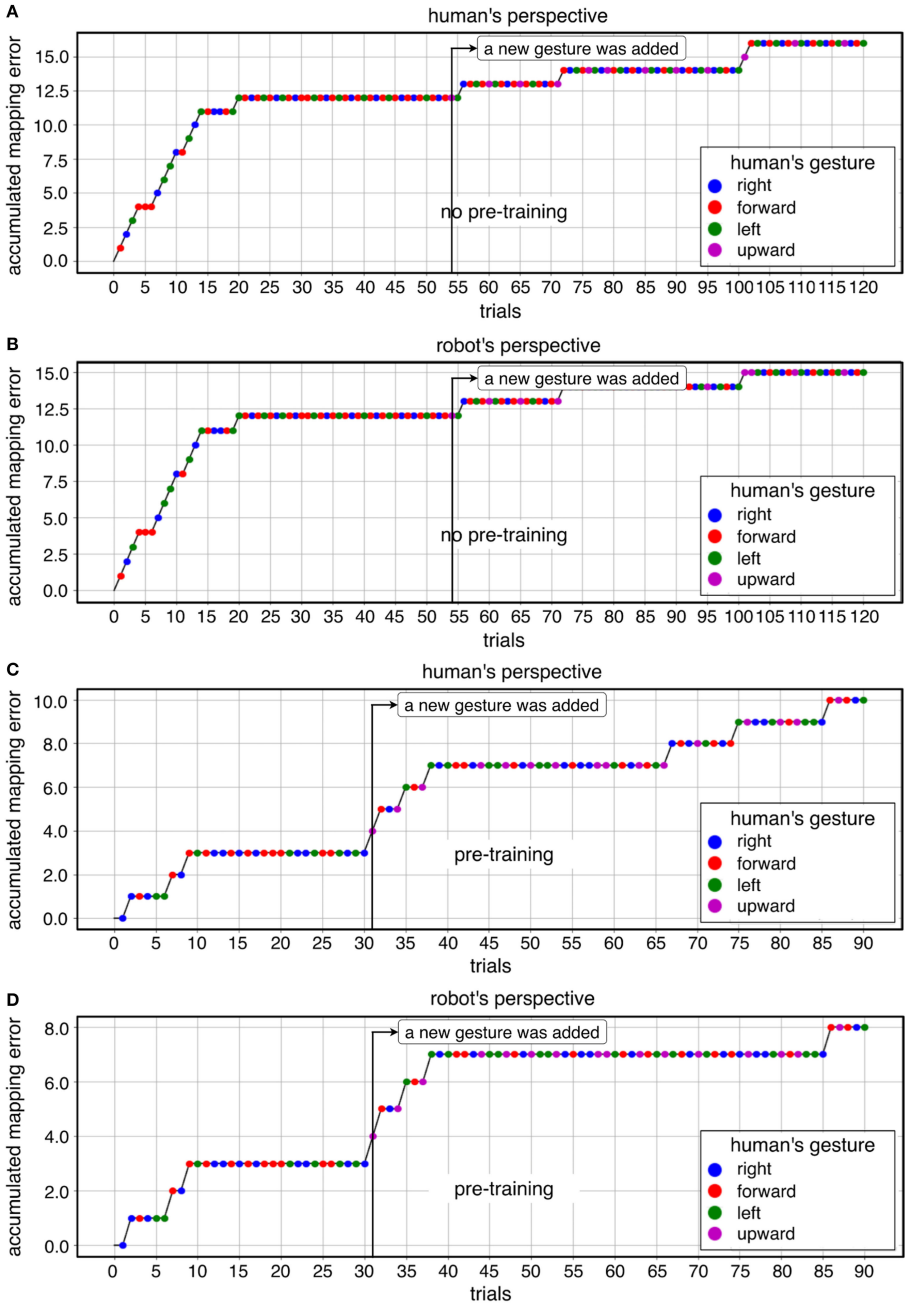
A descriptive visualization of learning progress for both learning conditions in both perspective: **(A)** cold-start learning (no pre-training) for human's perspective, **(B)** cold-start learning (no pre-training) for robot's perspective, **(C)** warm-start learning (pre-training) for human's perspective, **(D)** warm-start learning (pre-training) for robot's perspective.

**Figure 6:**
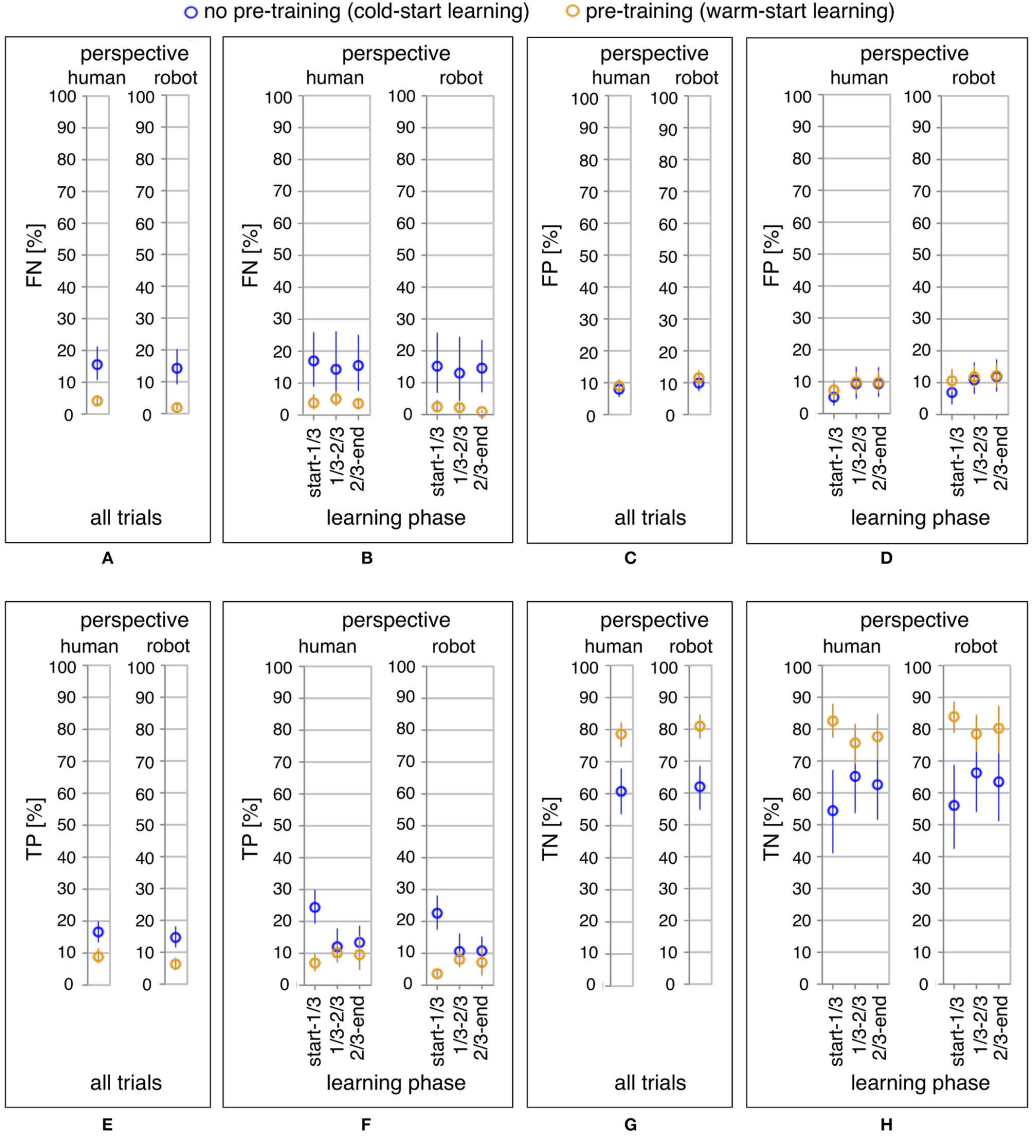
ErrP-classification performance (FN, FP, TP, TN): the average of FN across all datasets, the average of FP across all datasets, the average of TP across all datasets, the average of TN across all datasets for the whole learning phase **(A,C,E,G)** and the three learning phases **(B,D,F,H)** in both perspectives. For each perspective, both learning conditions are compared: pre-training (yellow) vs. no pre-training (blue).

Further, we compared both learning conditions and both perspectives for all trials to analyze effects of learning condition and perspective on mapping errors and ErrP-classification performance in the whole learning phase (see Figures 4A, 6A,C,E,G, a descriptive visualization of the whole learning phase as an example, see Figure 5). To this end, the results were pooled from three learning phases for each learning condition and each perspective. This is equivalent to a two-way repeated measures ANOVA with learning condition (2 levels: warm-start learning, cold-start learning) as between-subjects factor and perspective (2 levels: human's perspective, robot's perspective) as within-subjects factor. Two *post-hoc* analyses were performed, i.e., pairwise comparisons were performed at each factor to compare (1) both learning conditions for each perspective (human's perspective vs. robot's perspective) and (2) both perspectives for each learning condition (warm-start learning vs. cold-start learning). Bonferroni correction was performed for pairwise comparisons.

Finally, we performed three investigations to find out (a) relationship between robot's learning performance (mapping errors) and ErrP-classification performance (see Table 4A; a descriptive visualization, see **Figure 8A** and Supplementary Figure 1), (b) relationship between robot's learning performance (mapping errors) and gesture errors (see Table 4B; a descriptive visualization, see **Figure 8B**), and (c) relationship between gesture errors and ErrP-classification performance (see Table 4C; a descriptive visualization, see **Figure 8C** and Supplementary Figure 2). To this end, we calculated correlation coefficients for each investigation (a,b,c). Concerning ErrP-classification performance, we performed a correlation analysis separately for ErrP misclassifications (FN ∪ FP), TP, TN, FP, and FN (see Tables 4A,C). All correlation analyses were performed separately for each learning condition (warm-start learning, cold-start learning) and each perspective (human's perspective, robot's perspective). Correlation coefficients and significances were reported for each correlation analysis (see Table 4).

**Table 4:** Correlation analysis.

	**Human's perspective**		**Robot's perspective**	
	**Cold-start learning**	**Warm-start learning**	**Cold-start learning**	**Warm-start learning**
**(A) Correlation between robot's learning performance and ErrP-classification performance**				
ErrP misclassification	0.892^**^	0.684^**^	0.888^**^	0.680^**^
FN	0.927^**^	0.715^**^	0.944^**^	0.705^**^
FP	−0.172	0.453^*^	−0.262	0.584^**^
TN	−0.950^**^	−0.897^**^	−0.942^**^	−0.869^**^
TP	0.622^**^	0.934^**^	0.693^**^	0.965^**^
FNR (1-TPR)	0.707^**^	0.209	0.745^**^	0.228
FPR (1-TNR)	0.486^*^	0.577^**^	0.395	0.660^**^
**(B) Correlation between robot's learning performance and gesture errors**				
Gesture errors	−0.089	0.803^**^	−0.274	0.503^*^
**(C) Correlation between ErrP-classification performance and gesture errors**				
ErrP misclassification	−0.221	0.488^*^	−0.090	0.573^**^
FN	−0.190	0.604^**^	−0.248	0.356
FP	−0.036	0.272^*^	0.385	0.533^*^
TN	0.101	−0.676^**^	0.150	−0.595^**^
TP	0.169	0.735^**^	0.214	0.485^*^
FNR (1-TPR)	0.075	0.208	0.001	0.037
FPR (1-TNR)	0.153	0.388	0.103	0.595^**^

(A) Correlation between the robot's learning performance (mapping errors) and the ErrP-classification performance for both learning conditions and both perspectives. (B) Correlation between the robot's learning performance (mapping errors) and gesture errors for both learning conditions and both perspectives. (C) Correlation between the ErrP-classification performance and gesture errors for both learning conditions and both perspectives. Note that

** stands for significant level of p < 0.01 (2-sided) and

* *stands for significant level of p < 0.05 (2-sided). TPR = 1-FNR; TNR = 1-FPR. ErrP misclassification: FP ∪ FN*.

## 3. Results

Table 3 shows the overall results of descriptive analysis: the number of mapping errors (robot's learning performance), gesture errors, and ErrP misclassifications including false positive (FP) and false negative (FN) for both perspectives and both learning conditions. In addition, false positive rate (FPR) and false negative rate (FNR) were reported for both perspectives and both learning conditions. As mentioned earlier, the number of trials varied between subjects in online test sets. Thus, we calculated the number of mapping errors, gesture errors, and ErrP misclassifications in % (details, see section 2.3).

### 3.1. Learning Performance of the Robot

In our HRI scenario, the robot learns the mapping between human gestures and robot's actions, i.e., correct gesture-action pairs. Hence, the number of errors in the mapping between human gestures and robot's actions (i.e., mapping errors) was used as performance measure. Table 3 shows the number of mapping errors for both learning conditions and both perspectives.

Figure 4A shows the comparison of the total number of mapping errors (i.e., in the whole learning phase) between both learning conditions for each perspective. The number of mapping errors was significantly decreased in the warm-start learning condition (pre-training) compared to the cold-start learning condition (no pre-training) in both perspectives [*F*_1,35_ = 12.29, *p* < 0.002*, human perspective:* *p* < 0.003*, robot perspective:* *p* < 0.002]. For both learning conditions, the number of mapping errors was reduced in robot's perspective compared to human's perspective for both learning conditions [*F*_1,35_ = 25.98, *p* < 0.001*, cold-start learning:* *p* < 0.011*, warm-start learning:* *p* < 0.001*]*.

Figure 4B shows the comparison of three different learning phases in both learning conditions. We divided the whole learning phase in three learning phases according to the time point of when a new gesture (changes of human intents) was added. Different patterns of the learning process were observed between both learning conditions. The number of mapping errors was not significantly varied between learning phases in warm-start learning, whereas a significant reduction of mapping errors was observed between learning phases in cold-start learning [*F*_2,70_ = 3.63, *p* < 0.033]. This pattern was shown for both perspectives. In warm-start learning, the number of mapping errors was slightly (but not significantly) increased in the second learning phase (after adding a new gesture) and slightly (but not significantly) reduced in the third learning phase. [*human's perspective: start-1/3 vs. 1/3-2/3:* *p* = 0.51*, 1/3-2/3 vs. 2/3-end:* *p* = 1.0*, start-1/3 vs. 2/3-end:* *p* = 1.0*; robot's perspective: start-1/3 vs. 1/3-2/3:* *p* = 0.41*, 1/3-2/3 vs. 2/3-end:* *p* = 1.0*, start-1/3 vs. 2/3-end:* *p* = 1.0]. In cold-start learning, the number of mapping errors was significantly reduced in the second learning phase (after adding a new gesture) and slightly (but not significantly) increased in the third learning phase [*human's perspective: start-1/3 vs. 1/3-2/3:* *p* < 0.001*, 1/3-2/3 vs. 2/3-end:* *p* = 1.0*, start-1/3 vs. 2/3-end:* *p* < 0.01*; robot's perspective: start-1/3 vs. 1/3-2/3:* *p* < 0.001*, 1/3-2/3 vs. 2/3-end:* *p* = 1.0*, start-1/3 vs. 2/3-end:p* < 0.006]. Further, the number of mapping errors was significantly reduced for warm-start learning compared to cold-start learning in the first learning phase for both perspectives [*warm-start learning vs. cold-start learning:* *p* < 0.001 *for both perspectives*]. However, there was no significant difference between both learning conditions in the second learning phase [*warm-start learning vs. cold-start learning:* *p* = 0.079 *for human's perspective;* *p* = 0.051 *for robot's perspective*]. In the final learning phase, the number of mapping errors was again reduced for warm-start learning compared to cold-start learning [*warm-start learning vs. cold-start learning:* *p* < 0.022 *for human's perspective;* *p* < 0.010 *for robot's perspective*].

Figure 5 shows a descriptive visualization of the learning progress of the whole learning phase as an example, which was separately visualized in both learning conditions and both perspectives. In the beginning of the learning phase, we observed a high increase of mapping errors in cold-start learning compared to warm-start learning. Accordingly, the learning curve in cold start learning slowly stabilized compared to warm start learning before a new gesture was added. This learning pattern was shown for both perspectives. However, once the learning curve had stabilized, adding a new gesture to cold start learning had less impact on learning than warm-start learning. In contrast, the number of mapping errors has been increased immediately after adding a new gesture for warm-start learning (Figures 5C,D after 30 trials). After the learning curve had stabilized, there was some variation in both learning conditions. In the late learning phase (2/3-end) fluctuations were observed, which were caused by FP especially during warm start learning. In warm-start learning, FP occurred more frequently in the late learning phase compared to cold-start learning. This was consistent with the correlation analysis, according to which FP showed a significant correlation with mapping errors for the learning condition *warm start*, but not for the learning condition *cold start* (Table 4A, details, see section 3.3). Note that the class ratio was different depending on datasets as shown in Figures 5C,D, since the number of correct and wrong actions depends on the online learning performance of the robot.

In summary, it can be observed that the total number of mapping errors of the robot during warm-start learning has been reduced compared to cold-start learning in both perspectives. After adding a new gesture, the number of mapping errors in warm-start learning was slightly increased, while the number of mapping errors in cold-start learning was reduced after adding a new gesture. In warm start learning, an earlier stabilization of the robot's learning progress was observed than in cold-start learning at the beginning of the learning phases. That means, the learning curve was stabilized quickly in warm-start learning compared to cold-start learning. In other words, the algorithm is converged in warm-start learning before adding a new gesture, whereas the convergence was not reached in cold-start learning before adding a new gesture. However, the difference in mapping errors between the two learning conditions disappeared in the second learning phase (after adding a new gesture), because a slight increase in mapping errors in warm start learning and a significant reduction in mapping errors in cold start learning canceled out the effect of warm start learning on the robot's learning performance in the second learning phase. In fact, there were less fluctuations of learning progress for cold-start learning condition across all subjects after adding a new gesture compared to warm-start learning. Note that individual differences were more clearly observed for cold-start learning compared to warm-start learning (see **Figure 8**, details, see section 3.3).

### 3.2. ErrP-Classification Performance in the Whole Learning Phase

In our HRI scenario, the results of classifiers trained to recognize ErrPs were used as a reward in our learning algorithm. To measure the ErrP classification performance, a confusion matrix was calculated and the number of FN, FP, TP, and TN was used as performance metric.

Table 3 shows the number of FN, FP, TP, and TN. The number of FN was significantly reduced in warm-start learning compared to cold-start learning. However, the number of FP was slightly (but not statistically) increased for warm-start learning compared to cold-start learning. Hence, the number of FN was higher for FN than FP, whereas the number of FN was lower compared to FP in warm-start learning.

Figure 6 shows the comparison of ErrP-classification performance (FN, FP, TP, TN) between both learning conditions and both perspectives for all trials (Figures 6A,C,E,G). We found differences between both learning conditions in ErrP-detection performances. The number of ErrP misclassifications (FN ∪ FP) was reduced for warm-start learning compared to cold-start learning under both perspectives [*F*_1,35_ = 5.36, *p* < 0.029*, human perspective:* *p* < 0.031*, robot perspective:* *p* < 0.027]. Especially, the number of FN was substantially reduced in warm-start learning compared to cold-start learning under both perspectives [*F*_1,35_ = 7.21, *p* < 0.012*, human perspective:* *p* < 0.015*, robot perspective:* *p* < 0.01]. However, the number of FP was not significantly differed between both learning conditions. [*F*_1, 35_ = 0.034, *p* < 0.569*, human perspective:* *p* = 0.64*, robot perspective:* *p* = 0.53]. The number of TN was increased for warm-start learning compared to cold-start learning [*F*_1, 35_ = 9.29, *p* < 0.005*, human perspective:* *p* < 0.006*, robot perspective:* *p* < 0.005]. In contrast, the number of TP was increased for cold-start learning compared to warm-start learning [*F*_1, 35_ = 11.10, *p* < 0.003*, human perspective:* *p* < 0.006*, robot perspective:* *p* < 0.002]. The FNR was reduced for warm-start learning compared to cold-start learning in robot's perspective, but not in human's perspective [*F*_1, 35_ = 3.81, *p* = 0.059*, human perspective:* *p* = 0.227*, robot perspective:* *p* < 0.027]. The FPR was not significantly reduced for warm-start learning compared to cold-start learning under both perspectives [*F*_(1, 35)_ = 0.67, *p* = 0.420*, human perspective:* *p* = 0.391*, robot perspective:* *p* = 0.461].

We also found differences between both perspectives in ErrP-detection performances. Under both learning conditions, the number of aberrations in the robot perspective was reduced compared to the human perspective. [*F*_1, 35_ = 25.98, *p* < 0.001*, cold-start learning:* *p* < 0.010*, warm-start learning:* *p* < 0.001]. The number of FN was reduced under the robot perspective compared to the human perspective for warm start learning, but not for cold start learning [*F*_1, 35_ = 16.89, *p* < 0.002*, cold-start learning:* *p* = 0.06*, warm-start learning:* *p* < 0.001]. In contrast, the number of FP was increased under the robot perspective compared to the human perspective under both learning conditions [*F*_1, 35_ = 16.30, *p* < 0.001 *cold-start learning:* *p* < 0.023*, warm-start learning:* *p* < 0.003]. Altogether, the number of ErrP misclassifications (FN ∪ FP) was not differed between both perspectives [*F*_1, 35_ = 0.82, *p* = 0.372*, cold-start learning:* *p* = 0.39*, warm-start learning:* *p* = 0.69]. The number of TNs was increased from the robot perspective compared to the human perspective for warm start learning, but not for cold start learning [*F*_1, 35_ = 16.92, *p* < 0.001*, cold-start learning:* *p* = 0.058*, warm-start learning:* *p* < 0.001]. However, the number of TP from the robot perspective was reduced compared to the human perspective for both learning conditions [*F*_1, 35_ = 16.30, *p* < 0.001*, cold-start learning:* *p* < 0.02*, warm-start learning:* *p* < 0.002]. FNR was reduced from the robot perspective compared to the human perspective in warm start learning, but not in cold start learning [*F*_1, 35_ = 4.34, *p* < 0.046*, cold-start learning:* *p* = 0.058*, warm-start learning:* *p* < 0.02]. The FPR was increased from the robot perspective compared to the human perspective for both learning conditions [*F*_1, 35_ = 12.90, *p* < 0.002*, cold-start learning:* *p* < 0.032*, warm-start learning:* *p* < 0.008]. Note that we have not found any interaction between the three factors (learning condition, learning phase, perspective). Hence, the results of pairwise comparisons between levels of factors could be well-interpreted.

In summary, it can be said that the ErrP classification performance was influenced by the learning conditions. Especially wrong classifications of incorrect robot actions (FN) and correct classifications of correct robot actions (TN) were strongly influenced by the learning conditions. Correct classifications of erroneous actions (TP) were also influenced by the learning condition, but this effect was not higher than TN or FN.

### 3.3. Effect of ErrP-Classification Performance on Learning Performance

Figure 6 shows ErrP-classification performance (FN, FP, TP, TN) in the three learning phases (Figures 6B,D,F,H). As expected, we found that the pattern of TP and FN (i.e., correct or incorrect classifications of *erroneous actions* of the robot) was coherent with the pattern of *erroneous actions* of the robot (i.e., mapping errors) (Figure 4B vs. Figure 6F; Figure 4B vs. Figure 6B). However, the pattern of TN (i.e., correct classifications of *correct actions* of the robot) was reversed compared to the pattern of mapping errors (Figure 4B vs. Figure 6H).

#### 3.3.1. Correct Classifications of Erroneous Actions of the Robot (TP)

Figure 6F shows the number of TP for the three learning phases and both learning conditions. As expected, the pattern of correct classifications of *erroneous actions* of the robot matched with the pattern of *erroneous actions* of the robot (mapping errors). In warm-start learning, the number of TP was slightly (but not significant) increased in the second learning phase and slightly (but not significant) reduced in the third learning phase [*human's perspective: start-1/3 vs. 1/3-2/3:* *p* = 0.532*, 1/3-2/3 vs. 2/3-end:* *p* = 1.0*, start-1/3 vs. 2/3-end:* *p* = 1.0*; robot's perspective: start-1/3 vs. 1/3-2/3:* *p* = 0.155*, 1/3-2/3 vs. 2/3-end:* *p* = 0.556*, start-1/3 vs. 2/3-end:* *p* = 1.0]. In cold-start learning, the number of TP was significantly reduced in the second learning phase and slightly (but not significantly) increased in the third learning phase [*human's perspective: start-1/3 vs. 1/3-2/3:* *p* < 0.001*, 1/3-2/3 vs. 2/3-end:* *p* = 1.0*, start-1/3 vs. 2/3-end:* *p* < 0.004*; robot's perspective: start-1/3 vs. 1/3-2/3:* *p* < 0.001*, 1/3-2/3 vs. 2/3-end:* *p* = 1.0*, start-1/3 vs. 2/3-end:* *p* < 0.006]. The same pattern was observed in the learning performance of the robot, i.e., mapping errors (Figure 4B vs. Figure 6F). Furthermore, the number of TPs for warm start learning was reduced compared to cold start learning only for the beginning of the learning phase for both perspectives [*start-1/3: warm-start learning vs. cold-start learning:* *p* < 0.001 *in both perspectives*]. After adding a new gesture, there was no significant difference between both learning conditions [*1/3-2/3: warm-start learning vs. cold-start learning;* *p* = 0.534 *in human's perspective*, *p* = 0.417 *in robot's perspective; 2/3-end: warm-start learning vs. cold-start learning:* *p* = 0.334 *in human's perspective*, *p* = 0.305 *in robot's perspective*]. In cold-start learning, differences between the two perspectives only became evident for the final learning phase [*human's perspective vs. robot's perspective:* *p* = 0.153 *for start-1/3;* *p* = 0.178 *for 1/3-2/3;* *p* < 0.012 *for 2/3-end*]. In warm-start learning, differences between both perspectives were found in all learning phases [*human's perspective vs. robot's perspective:* *p* < 0.007 *for start-1/3;* *p* < 0.010 *for 1/3-2/3;* *p* < 0.013 *for 2/3-end*].

#### 3.3.2. Correct Classifications of Correct Actions of the Robot (TN)

Figure 6H shows the number of TN for three learning phases and both learning conditions. As expected, we observed that the pattern of learning phases in TN was inverse to the pattern of learning phases in mapping errors (Figure 4B vs. Figure 6H). In warm-start learning, the number of TN was slightly (but not significant) reduced in the second learning phase and slightly (but not significant) increased in the third learning phase for both perspectives [*human's perspective: start-1/3 vs. 1/3-2/3:* *p* = 0.102*, 1/3-2/3 vs. 2/3-end:* *p* = 0.712*, start-1/3 vs. 2/3-end:* *p* = 1.0*; robot's perspective: start-1/3 vs. 1/3-2/3:* *p* = 1.0*, 1/3-2/3 vs. 2/3-end:* *p* = 0.251*, start-1/3 vs. 2/3-end:* *p* = 1.0]. In contrast, in cold-start learning, the number of TN was significantly increased in the second learning phase and slightly (but not significantly) reduced in the third learning phase for both perspectives [*human's perspective: start-1/3 vs. 1/3-2/3:* *p* < 0.001*, 1/3-2/3 vs. 2/3-end:* *p* = 1.0*, start-1/3 vs. 2/3-end:* *p* < 0.001*; robot's perspective: start-1/3 vs. 1/3-2/3:* *p* < 0.012*, 1/3-2/3 vs. 2/3-end:* *p* = 1.0*, start-1/3 vs. 2/3-end:p* = 0.321]. In particular, the number of TN for warm start learning was increased compared to cold start learning. This was only shown for the initial phase and the end of the learning phase from both perspectives [*start-1/3:* *p* < 0.001 *in both perspectives; 1/3-2/3:* *p* = 0.116 *in human's perspective*, *p* = 0.072 *in robot's perspective; 2/3-end:* *p* < 0.039 *in human's perspective*, *p* < 0.022 *in robot's perspective*]. Further, we found differences between both perspectives in the second and final learning phase for warm-start learning and only in the beginning of learning phase in cold-start learning [*warm-start learning:* *p* = 0.055 *for start-1/3;* *p* < 0.002 *for 1/3-2/3;* *p* < 0.007 *for 2/3-end; cold-start learning:* *p* < 0.033 *for start-1/3;* *p* = 0.190 *for 1/3-2/3;* *p* = 0.371 *for 2/3-end*].

#### 3.3.3. Incorrect Classifications of Correct Actions of the Robot (FP)

Figure 6D shows the number of FP for the three learning phases under both learning conditions. We observed no difference between learning phases and between learning conditions. We found no significant difference between three learning phases in both perspective [*(a) human's perspective: warm-start learning: start-1/3 vs. 1/3-2/3:* *p* = 0.168*, 1/3-2/3 vs. 2/3-end:* *p* = 0.216*, start-1/3 vs. 2/3-end:* *p* = 1.0*, cold-start learning: start-1/3 vs. 1/3-2/3:* *p* = 0.084*; 1/3-2/3 vs. 2/3-end:* *p* = 0.313*; start-1/3 vs. 2/3-end:* *p* = 1.0*; (b) robot's perspective: warm-start learning: start-1/3 vs. 1/3-2/3:* *p* = 1.0*, 1/3-2/3 vs. 2/3-end:* *p* = 1.0*, start-1/3 vs. 2/3-end:* *p* = 1.0*, cold-start learning: start-1/3 vs. 1/3-2/3:* *p* = 0.438*, 1/3-2/3 vs. 2/3-end:* *p* = 0.917*, start-1/3 vs. 2/3-end:* *p* = 0.438]. Further, we found no differences between both learning conditions for both perspectives [*human's perspective: warm-start learning vs. cold-start learning:* *p* = 0.323 *for start-1/3*, *p* = 0.867 *for 1/3-2/3*, *p* = 0.891 *for 2/3-end; robot's perspective: warm-start learning vs. cold-start learning:* *p* = 0.323 *for start-1/3*, *p* = 0.867 *for 1/3-2/3*, *p* = 0.891 *for 2/3-end*]. Further, we found significant differences between both perspectives for all learning phases in warm-start learning [*human's perspective vs. robot's perspective:* *p* < 0.008 *for start-1/3;* *p* < 0.014 *for 1/3-2/3:* *p* < 0.011 *for 2/3-end*]. In cold-start learning, differences between both perspectives were shown only for the final learning phase [*human's perspective vs. robot's perspective:* *p* = 0.164 *for start-1/3;* *p* = 0.067 *for 1/3-2/3:* *p* < 0.015 *for 2/3-end*].

#### 3.3.4. Incorrect Classifications of Erroneous Actions of the Robot (FN)

Figure 6B shows the number of FN for the three learning phases under both learning conditions. Only in cold-start learning, the pattern of FN was coherent with the pattern of mapping errors. We found no significant difference between three learning phases for both perspectives [*(a) human's perspective: warm-start learning: start-1/3 vs. 1/3-2/3:* *p* = 0.964*, 1/3-2/3 vs. 2/3-end*, *p* = 1.0*; start-1/3 vs. 2/3-end:* *p* = 1.0*; cold-start learning: start-1/3 vs. 1/3-2/3:* *p* = 0.835*, 1/3-2/3 vs. 2/3-end:* *p* = 1.0*, start-1/3 vs. 2/3-end:* *p* = 1.0*; (b) robot's perspective: warm-start learning: start-1/3 vs. 1/3-2/3:* *p* = 1.0*, 1/3-2/3 vs. 2/3-end:* *p* = 1.0*, start-1/3 vs. 2/3-end:* *p* = 1.0*; cold-start learning: start-1/3 vs. 1/3-2/3:* *p* = 1.0*, 1/3-2/3 vs. 2/3-end:* *p* = 1.0*, start-1/3 vs. 2/3-end:* *p* = 1.0]. Further, the number of FN was reduced for warm-start learning compared to cold-start learning for the first and the final learning phase, but not for the second learning phase. This pattern was shown for both perspectives [*human's perspective: warm-start learning vs. cold-start learning:* *p* < 0.011 *for start-1/3*, *p* = 0.051 *for 1/3-2/3*, *p* < 0.002 *for 2/3-end; robot's perspective: warm-start learning vs. cold-start learning:* *p* < 0.008 *for start-1/3*, *p* = 0.085 *for 1/3-2/3*, *p* = 0.009 *for 2/3-end*]. Further, we found significant differences between both perspectives for the second and the final learning phase, but not for the first learning phase in warm-start learning [*human's perspective vs. robot's perspective:* *p* = 0.060 *for start-1/3*, *p* < 0.002 *for 1/3-2/3*, *p* < 0.007 *for 2/3-end*]. The reversed pattern was shown in cold-start learning [*human's perspective vs. robot's perspective:* *p* < 0.025 *for start-1/3*, *p* = 0.135 *for 1/3-2/3*, *p* = 0.371 *for 2/3-end*].

#### 3.3.5. Correlation Between ErrP-Classification Performance and Mapping Errors

Table 4A shows the correlation between learning performance of the robot (mapping errors) and ErrP-classification performance for each learning condition and each perspective, in which correlations of mapping errors were separately calculated with ErrP misclassifications (FP∪FN),TP, TN, FP, and FN (details for statistical analysis, see section 2.5). Note that ^**^ stands for significant level of *p* < 0.01 (2-sided) and ^*^ stands for significant level of *p* < 0.05 (2-sided). **Figure 8A** shows a descriptive visualization of robot's learning performance and ErrP misclassification (more details, see Supplementary Figure 1, which shows a descriptive visualization of each correlation shown in Table 4A). An individual dot represents the result of mapping errors corresponding to ErrP misclassifications (FP ∪ FN), TP, TN, FP, and FN in each dataset, where different colors (yellow, blue) represent different learning conditions (warm-start learning, cold-start learning).

As expected, we observed a high correlation between learning performance of the robot and ErrP-classification performance under both learning conditions (see Table 4A). However, a higher correlation was observed for cold-start learning compared to warm-start learning. This pattern was more obviously shown in FN and TN. FN strongly correlated with learning performance in the cold-start learning condition compared to the warm-start learning condition [*cold-start learning vs. warm-start learning:* *r* = 0.927 *vs*. *r* = 0.715 *for human's perspective; cold-start learning vs. warm-start learning:* *r* = 0.944 *vs*. *r* = 0.705 *for robot's perspective*]. Note that we obtained a single correlation coefficient for each correlation analysis. Hence, the comparison between learning conditions was descriptively reported. The same pattern was shown for TN [*cold-start learning vs. warm-start learning:* *r* = −0.950 *vs*. *r* = −0.897 *for human's perspective; cold-start learning vs. warm-start learning:* *r* = −0.942 *vs*. *r* = −0.869 *for robot's perspective*]. In contrast, the reversed pattern was shown for TP, i.e., a higher correlation was observed for the warm-start learning compared to cold-start learning [*cold-start learning vs. warm-start learning:* *r* = 0.622 *vs*. *r* = 0.934 *for human's perspective; cold-start learning vs. warm-start learning:* *r* = 0.693 *vs*. *r* = 0.965 *for robot's perspective*]. For FP, there was no correlation for cold-start learning [*human's perspective:* *r* = −0.172*, robot's perspective:* *r* = −0.262].

Further, a descriptive analysis showed that a higher difference between datasets in robot's learning performance was observed in cold-start learning compared to warm-start learning. As shown in **Figure 8A**, all datasets of warms-start learning were placed in the dark green boxes, whereas 5 datasets of cold-start learning were placed in the light green boxes. The same pattern was shown in ErrP-detection performance (**Figure 8A**), which was a plausible reason for a high correlation between robot's learning performance and ErrP-detection performance (Table 4A). Note again that an individual dot represents the result of mapping errors corresponding to ErrP misclassifications (FP ∪ FN), TP, TN, FP, and FN in each dataset and different colors (yellow, blue) represent different learning conditions (warms-start learning and cold-start learning).

In summary, FN had a stronger impact on cold-start learning compared to warm-start learning, whereas FP had a stronger effect on warm-start learning compared to cold-start learning. In other words, the learning performance of the robot was impaired more during cold start learning than during warm start learning if incorrect robot actions were not detected, i.e., ErrPs were not detected if the actions of the robot were wrong. Further, FN had an effect on learning performance for both learning conditions, whereas FP had an impact on learning performance for warm-start learning, but not for cold-start learning. Consistent with a higher number of mapping errors, the number of TP was higher in cold-start learning than in warm-start learning.

### 3.4. Effect of Gesture Errors on Learning Performance

As mentioned earlier, we considered wrong recordings of human gestures as gesture errors, which lead to incoherences between performed and perceived gestures, i.e., incoherences between gestures performed by the subjects and gestures recorded by LeapMotion. The subjects perceived their own performed gesture and the robot perceived the gesture features recorded by LeapMotion. Therefore we analyzed the learning performance of robots and the ErrP classification performance depending on the two perspectives (robot perspective/human perspective).

**Figure 7:**
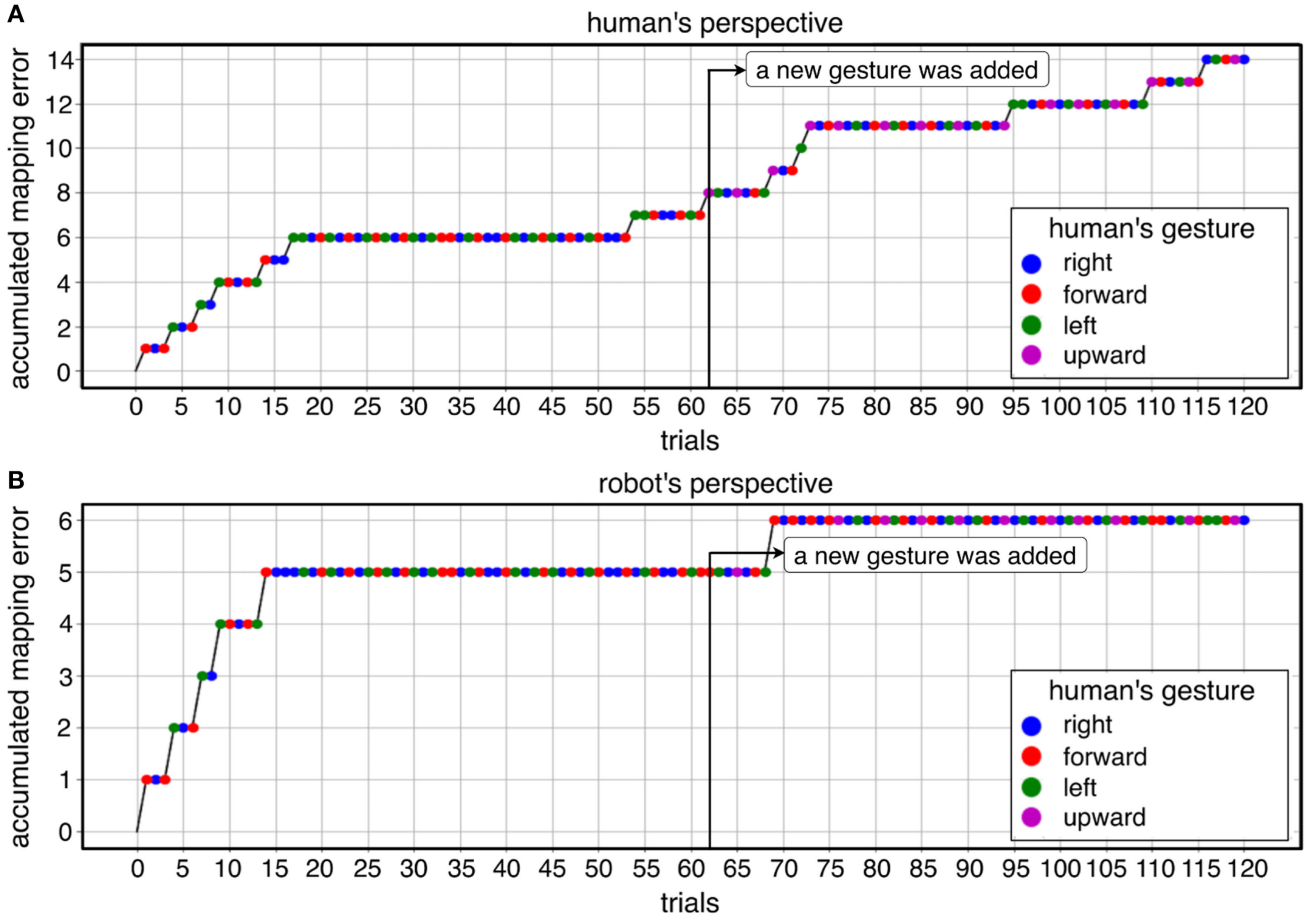
A descriptive analysis of differences in learning progress between both perspectives (**A** and **B**) that are caused by gesture errors. A descriptive visualization of learning progress of both perspectives is shown in the cold-start learning condition (no pre-training) as an example of one subject. The first five mapping errors (trial 1, 2, 4, 7, 9, 14) and the mapping errors in the trial 69 are the same for both perspectives. Other mapping errors (trial 17, 54, 62, 72, 73, 95, 110, 116) are shown for the human's perspective, but not for robot's perspective. Such different perceptions between human and robot due to gesture errors and their impacts on learning progress are analyzed in consideration of interaction with ErrP-detection performance and summarized in Table 5 (details, see text).

Figure 7 shows differences in learning progress between both perspectives that are caused by gesture errors. Gestures that were performed by the subjects are depicted in Figure 7A, whereas gestures that were recorded by the Leap Motion and perceived by the robot are depicted in Figure 7B. As shown in Figure 7, gestures were differently colored depending on perspective, e.g., *upward* (violet point) for human's perspective and *forward* (red point) for robot's perspective on the same action of the robot in the trial 69 (Figure 7A vs. Figure 7B, see Table 5D). When there were no gesture errors, wrong actions of the robot (mapping errors) were the same for both perspectives (see trial 1, 2, 4, 7, 9, 14 in Figure 7). When gesture errors occurred, the effect of gesture errors was not clear, which required a further analysis (details, see Table 5).

**Table 5:** Different perceptions between humans and robots due to gesture errors (9 gesture errors in total in this example, see cases C and D).

**Case**	**Trial**	**Human**	**Recorded**	**Robot's**	**ErrP**	**Human**	**Robot**
		**gesture**	**gesture**	**action**	**detection**	**CL**	**CL**
A	5	Right	Right	Right	No ErrP	TN	TN
	6	Forward	Forward	Forward	No ErrP	TN	TN
	8	Right	Right	Right	No ErrP	TN	TN
	9	Left	Left	Forward	ErrP	TP	TP
	10	Forward	Forward	Forward	No ErrP	TN	TN
	11	Right	Right	Right	No ErrP	TN	TN
	12	Forward	Forward	Forward	No ErrP	TN	TN
	13	Left	Left	Forward	ErrP	TP	TP
	15	Right	Right	Right	No ErrP	TN	TN
	16	Right	Right	Right	No ErrP	TN	TN
B	1	Forward	Forward	Right	ErrP	TP	TP
	2	Right	Right	Right	No ErrP	TN	TN
	3	Forward	Forward	Forward	No ErrP	TN	TN
	4	Left	Left	Forward	No ErrP	FN	FN
	7	Left	Left	Forward	No ErrP	FN	FN
	14	Forward	Forward	Left	ErrP	TP	TP
C	17	Left	Right	Right	No ErrP	FN	TN
	54	Left	Right	Right	ErrP	TP	FP
	62	Upward	Forward	Forward	ErrP	TP	FP
	72	Left	Right	Right	No ErrP	FN	TN
	73	Upward	Forward	Forward	ErrP	TP	FP
	95	Left	Right	Right	ErrP	TP	FP
	110	Upward	Forward	Forward	ErrP	TP	FP
	116	Right	Left	Left	ErrP	TP	FP
D	69	Upward	Forward	Right	ErrP	TP	TP

*The learning progress of this example is shown in Figure 7. Four cases of interaction between gesture errors and ErrP recognition performance and their effects on learning progress were observed: (A) No occurrence of gesture errors and correct actions of the robot from both perspectives, (B) No occurrence of gesture errors and wrong actions of the robot from both perspectives, (C) occurrence of gesture errors and correct actions of the robot from the robot's perspective, but not from the human's perspective, and (D) occurrence of gesture errors and wrong actions of the robot from both perspectives. CL: classification performance. robot CL: ErrP-detection performance from the robot's perspective. human CL: ErrP-detection performance from the human's perspective. Note that the robot's perception (robot CL) affects learning progress and the elicitation of ErrPs is based on the human's perception (matching between human gesture and robot's action). Note that not all trials are described in this example*.

Table 5 shows four cases where we observed the interaction effects of gesture errors on learning performance (the correctness of robot actions, i.e., mapping errors) with ErrP recognition performance: (A) No occurrence of gesture errors and correct actions of the robot for both perspectives, (B) No occurrence of gesture errors and wrong actions of the robot for both perspectives, (C) Occurrence of gesture errors and correct robot actions from the robot's perspective, but incorrect robot actions from the human perspective, and (D) Occurrence of gesture errors and wrong actions of the robot from both perspectives. Note that the trials that are visualized in Figure 7 are equivalent to the trials that are shown in Table 5.

#### 3.4.1. Correct Actions of the Robot Without Gesture Errors (Table 5A)

When there were no gesture errors and the robot's actions were also correct, ErrP-detection performance had a direct impact on the learning process (Table 5A). In other words, the learning performance was affected only by ErrP-classification performance. Correct classifications, i.e., detections of *ErrPs* on wrong gesture-action pairs (TP) and detections of *No ErrPs* on correct gesture-action pairs (TN) had a positive impact on the learning process.

#### 3.4.2. Wrong Actions of the Robot Without Gesture Errors (Table 5B)

If the robot's actions were wrong even though there were no gesture errors, the learning performance was also only affected by the ErrP recognition performance (Table 5B). Correct classifications, i.e., detections of *ErrPs* on wrong gesture-action pairs (TP) and detections of *No ErrPs* on correct gesture-action pairs (TN) had a positive impact on the learning process. In contrast, wrong classifications, i.e., detections of *No ErrPs* on wrong gesture-action pairs (FN) and detections of *ErrPs* on correct gesture-action pairs (FP) had a negative effect on the learning process.

#### 3.4.3. Correct Actions of the Robot With Gesture Errors (Table 5C)

If the robot's actions were correct, although gesture errors occurred, we observed two different effects: (1) Gesture errors had a negative effect on the learning performance, when ErrP detection was correct from the human's perspective (trial 54, 62, 73, 95, 110, 116 in Table 5C) and (2) Negative effects of gesture errors were canceled out, when ErrP detection were wrong from the human's perspective (trial 17, 72 in Table 5C). For example, when ErrPs were detected on wrong gesture-action pairs from the human's perspective (e.g., *left*-*right* pair in trial 54 in Table 5C), ErrP classifications were correct (TP). In contrast, when the robot perceived correct gesture-action pairs on the same actions of the robot (e.g., *right*-*right* pair in trial 54 in Table 5C), ErrP classifications were wrong from the robot's perspective (FP), which led to negative impacts on the learning progress. However, such negative effects of gesture errors on learning performance were canceled out, when ErrP detections were wrong from the human's perspective. For example, ErrP classifications (detections of *No ErrP*) were wrong on gesture-action pairs (*left*-*right* pairs) from the human's perspective, whereas ErrP classifications (detections of *No ErrP*) were correct on gesture-action pairs (*right*-*right* pairs) from the robot's perspective (see trial 17, 72 in Table 5C). In this case, gesture errors had a positive effect on learning performance because the ErrPs recognition was incorrect from the human perspective.

#### 3.4.4. Wrong Actions of the Robot With Gesture Errors (Table 5D)

When gesture errors occurred and the robot's actions were wrong, the learning performance was affected only by ErrP-detection performance. In this case, gesture-action pairs were wrong from both perspectives (see trial 69 in Table 5D): *upward*-*right* pair for human's perspective and *forward*-*right* pair for robot's perspective. Hence, ErrP classifications (detections of *ErrPs*) were correct (TP) and learning performance was not negatively affected.

In general, the number of gesture errors varied between subjects and sets. We visualized two examples for different numbers of gesture errors: 9 gesture errors (Figures 7A,B, and Table 5) vs. one gesture error (Figures 5A,B, and Table 6).

**Table 6:** Different perceptions between human and robot due to gesture errors (one gesture error in total in this example, see case D).

**Case**	**Trial**	**Human**	**Recorded**	**Robot's**	**ErrP**	**Human**	**Robot**
		**gesture**	**gesture**	**action**	**detection**	**CL**	**CL**
A	5	Forward	Forward	Forward	No ErrP	TN	TN
	6	Forward	Forward	Forward	No ErrP	TN	TN
	9	Left	Left	Left	No ErrP	TN	TN
	11	Forward	Forward	Forward	No ErrP	TN	TN
	15	Forward	Forward	Forward	No ErrP	TN	TN
	16	Right	Right	Right	Right	TN	TN
	17	Right	Right	Right	Right	TN	TN
	18	Forward	Forward	Forward	Forward	TN	TN
	19	Left	Left	Left	Left	TN	TN
B	1	Forward	Forward	Left	ErrP	TP	TP
	2	Right	Right	Left	ErrP	TP	TP
	3	Left	Left	Right	No ErrP	FN	FN
	4	Forward	Forward	Right	ErrP	TP	TP
	7	Right	Right	Forward	ErrP	TP	TP
	8	Left	Left	Right	ErrP	TP	TP
	10	Right	Right	Left	ErrP	TP	TP
	12	Left	Left	Forward	ErrP	TP	TP
	13	Right	Right	Left	ErrP	TP	TP
	14	Left	Left	Forward	ErrP	TP	TP
	20	Left	Left	Forward	ErrP	TP	TP
	56	Right	Right	Upward	ErrP	TP	TP
	72	Forward	Forward	Left	ErrP	TP	TP
	102	Forward	Forward	Upward	ErrP	TP	TP
C	–	–	–	–	–	–	–
D	101	Upward	Forward	Right	ErrP	TP	TP

*Learning progress of this example is shown in Figures 5A,B. Four cases of the interaction between gesture errors and their impacts on learning progress were observed: (A) No occurrence of gesture errors and correct actions of the robot from both perspectives, (B) No occurrence of gesture errors and wrong actions of the robot from both perspectives, (C) occurrence of gesture errors and correct actions of the robot from the robot's perspective, but not from the human's perspective, and (D) occurrence of gesture errors and wrong actions of the robot from both perspectives. CL: classification performance. robot CL: ErrP-detection performance from the robot's perspective. human CL: ErrP-detection performance from the human's perspective. Note that the robot's perception (robot CL) affects learning progress and the elicitation of ErrPs is based on the human's perception (matching between human gesture and robot's action). Note that not all trials are described in this example*.

#### 3.4.5. Correlation Between Gesture Errors and Mapping Errors and Correlation Between Gesture Errors and ErrP-Classification Performance

**Figure 8:**
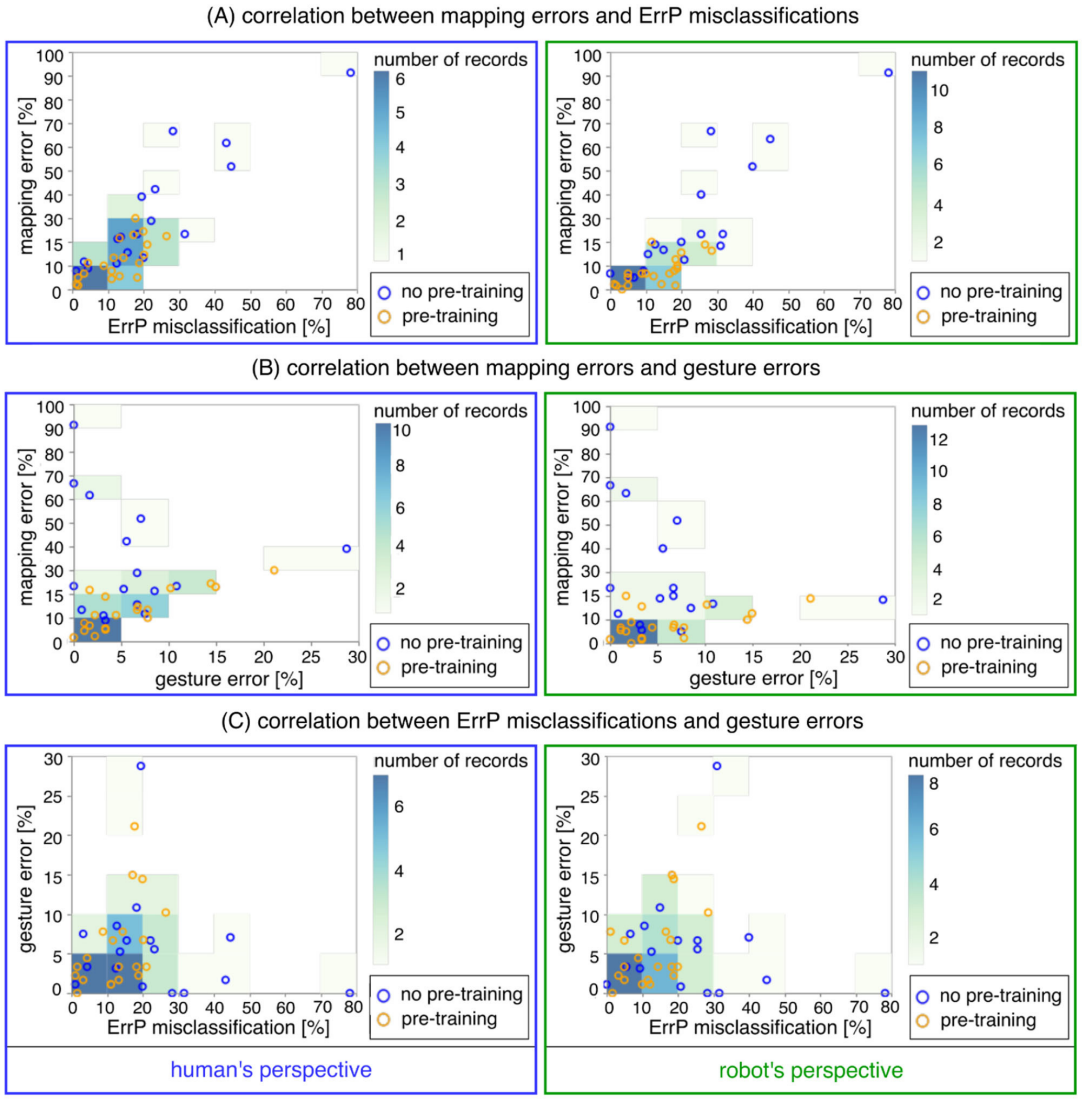
A descriptive visualization of correlation analysis: **(A)** correlation between mapping errors and ErrP misclassifications, **(B)** correlation between mapping errors and gesture errors, and **(C)** correlation between ErrP misclassifications and gesture errors. A statistical analysis of three types of correlation **(A–C)** is reported in Table 4. For each perspective, the comparison between both learning conditions is depicted in different colors: pre-training (yellow) vs. no pre-training (blue). Each dot represents each dataset (details, see text). A descriptive visualization of further correlation analyses are depicted in Supplementary Figures 1, 2.

Table 4B shows the correlation between gesture errors and mapping errors for both learning conditions and both perspectives and its descriptive visualization is shown in Figure 8B (more details, see Supplementary Figure 2). We found a correlation between gesture errors and the robot's learning performance for warm-start learning but not for cold-start learning [*cold-start learning vs. warm-start learning:* *r* = 0.803 *vs*. *r* = −0.089 *for human's perspective; cold-start learning vs. warm-start learning:* *r* = 0.503 *vs*. *r* = −0.274 *for robot's perspective*]. Furthermore, the reason why the correlation between gesture and mapping errors was only shown for warm start learning can be explained by further correlation analysis. Table 4C shows the correlation between ErrP-detection performance and gesture errors for both learning conditions and both perspectives and its descriptive visualization is shown in Figure 8C (more details, see Supplementary Figure 2). For both perspectives, we found a correlation between ErrP misclassifications and gesture errors for warm-start learning, but not for cold-start learning [*cold-start learning vs. warm-start learning:* *r* = −0.221 *vs*. *r* = 0.488 *for human's perspective; cold-start learning vs. warm-start learning:* *r* = −0.090 *vs*. *r* = 0.573 *for robot's perspective*]. Both correlation analyses (Tables 4B,C) showed that gesture errors had an impact on learning performance (mapping errors), only when gesture errors correlate with ErrP misclassifications (Table 4B vs. Table 4C, Figure 8B vs. Figure 8C).

In summary, it can be said that gesture errors affected the learning performance of the robot in other ways. Due to gesture errors, an incorrect feedback (human evaluation) was sent to the robot, although the human evaluation itself was correct. However, such negative effects of gesture errors on robot learning performance disappeared if the ErrP classification was incorrect. Furthermore, we could find out afterwards that gesture errors had no effect on the robot's learning performance if the robot action selection was wrong and the ErrP classification was correct.

### 3.5. Summary of Results

We showed that the robot learned actions that were best assigned to human gestures based on EEG-based reinforcement signals. In the proposed HRI scenario, human gestures were not predefined, i.e., no initial semantics of gestures was given to the robot. Rather, the robot learned the current meaning of human gesture (i.e., the meaning of human gesture that can be changed online). To this end, we used a contextual bandit approach that maximizes the expected payoff by updating the current human intention (human gesture) and the current human feedback (ErrP) after each action selection of the robot.

Robot learning and its online adaptation were successful for both warm-start learning and cold-start learning. Only for one subject robot learning was not successful in cold-start learning due to a very low detection performance of ErrPs used for human's intrinsic feedback (rewards). Further, cold-start learning required more data to reach a stabilization of the learning curve compared to warm-start learning *before* adding a new context (e.g., before adding a new gesture). However, cold-start learning was less affected by changes of the current context (e.g., *after* adding a new gesture) compared to warm-start learning, which indicates that cold-start learning was stable for updating of the learned strategy once learning reached convergence.

Online detection of ErrPs used for rewards in the used learning algorithm was successful for both learning conditions except for one subject who showed a very low performance of ErrP detections in cold-start learning. Our assumption that the ErrP-classification performance affects robot's learning performance was supported by a high correlation between robot's learning performance and ErrP-detection performance in both learning condition (Table 4A). Further, a descriptive analysis showed a higher variability between datasets in cold-start learning compared to warm-start learning, which can be shown in Figure 8. For example, five datasets of cold-start learning were placed in the light green boxes, whereas all datasets of warms-start learning were placed in the dark green boxes (Figure 8). However, correlation coefficients were computed for each learning condition and the comparison between both learning conditions (inference statistics) was not possible, since there was only one coefficient value for each learning condition.

Gesture errors that were not detected online but analyzed offline had no direct impact on robot's learning performance. Rather, gesture errors affected robot's learning performance only when gesture errors interacted with ErrP-detection performance. Especially, we observed a correlation between gesture errors and ErrP-detection performance in warm-start learning (Table 4C), which led to a correlation between robot's learning performance and gesture errors in warm-start learning (Table 4B). In contrast, we observed no correlation between gesture errors and ErrP-detection performance in cold-start learning (Table 4C), which resulted in *no* correlation between robot's learning performance and gesture errors in cold-start learning (Table 4B).

## 4. Discussion

In this paper, we analyzed errors that occur in HRI and their impacts on online learning performance of the robot. Our results indicate that a little prior knowledge facilitates learning progress and allows a faster stabilization of the learning curve compared to learning without prior knowledge. Warm-start learning was advantaged, since a few trials (i.e., gesture-action pairs) were pre-trained with the perfect human feedback (correct detections of ErrP/No ErrP). Further, the reason for the faster learning can be explained by the higher ErrP classification performance, i.e., the significant reduction of ErrP misclassifications in warm-start learning compared to cold-start learning. Especially the role of the FN, i.e., the absence of robot mistakes (mapping error), seems to be very important for learning performance both for learning with prior knowledge and for learning without prior knowledge. In contrast, false alarm (FP) seems to have a small overall effect on the robot's learning performance with a greater effect on warm-start learning compared to cold-start learning. This is supported by correlations between FN and mapping errors in both learning conditions and correlations between FP and mapping errors shown for warm-start learning but not for cold-start learning (Table 4A, Supplementary Figure 1). The reason why FN had a higher influence on the learning performance of the robot compared to FP can be explained by the use of different weights of rewards depending on the results of ErrP classifications (Table 2): our HRI scenario was designed that the predictions of correct mappings (No ErrP) were highly rewarded compared to the predictions of wrong mappings (ErrP), since a *correct* gesture-action pair should be learned by the UCB algorithm. Further, our results suggest that not only ErrP misclassifications (FN, FP) but also correct classifications of ErrPs/No ErrPs (TP, TN) can have an impact on learning performance of the robot under both learning conditions. This is supported by the findings of negative correlations of TN with mapping errors and positive correlations of TP with mapping error (Table 4A, Supplementary Figure 1). Further, the faster stabilization of the learning curve in warm-start learning seems to cause the lower number of TPs (correct detections of erroneous actions of the robot) in warm-start learning compared to cold-start learning, although the number of TNs (correct detections of correct action of the robot) in warm-start learning was higher compared to cold-start learning. Another possible reason why the ErrP classification performance was higher for warm-start learning compared to cold-start learning is that the subjects started always with warm-start learning before cold-start learning and thus the subjects could be more tired in cold-start learning compared to warm-start learning. The effect of tiredness on ErrP expression is relevant for continuous interaction and learning and will be investigated in future.

Our results indicate that learning without prior knowledge requires more trials to stabilize the learning curve compared to warm-start learning. This can be shown in the learning curve descriptively (e.g., Figure 5). However, cold-start learning was less affected by changes of the current context (e.g., *after* adding a new gesture) compared to warm-start learning, once learning reached convergence. This was shown by the result that the mean number of incorrect robot actions over all subjects was even statistically reduced during cold-start learning, although a new gesture was added to online learning (Figure 4). The reason why the number of erroneous actions of the robot was increased after adding a new gesture in warm-start learning in contrast to cold-start learning can be explained in the following way. For both learning conditions (warm-start learning/cold-start learning), the new gesture (*upwards*) was not chosen before and thus had a high variance, i.e., a high upper confidence interval (UCI), which leads to a high expected payoff, i.e., a high upper confidence bound (UCB) accordingly. In cold-start learning, the expected payoff of the previous learned gesture-action pairs (*left, right, forward*) could not be higher compared to the expected payoff of the new gesture (*upwards*) before adding the new gesture. Thus, for example, when the subject adds a new gesture, the probability that the new gesture is chosen could be high due to a high expected payoff caused by a high variance. That means, the transition to the learning of the new gesture could be very smoothy due to a high UCB caused by a high UCI. Thus, the algorithm could explore in a natural way. In contrast, the expected payoff of previous learned gesture-action pairs (*left, right, forward*) could be substantially higher compared to a new gesture-action pair (*upwards*) in warm-start learning. Hence, the algorithm could have no soft transition to the learning of the new gesture-action pair in warm-start learning. In fact, the expected payoff of three gesture-action pairs (*left, right, forward*) could be already high, since the UCB algorithm could reach very quickly convergence due to pre-training before adding the new gesture. For this reason, the transition to the learning of a new gesture-action pair could not be smoothly in warm-start learning, which could lead to the increased number of erroneous actions of the robot in warm-start learning immediately after adding a new gesture (Figure 4).

Our assumption that ErrP-classification performance used as rewards affects learning performance of the robot was confirmed by a high correlation between ErrP-classification performance and robot's learning performance in both learning conditions. However, gesture errors had an impact on robot' learning performance, only when gesture errors correlated with ErrP-classification performance. This indicates that gesture errors have an indirect effect on learning performance of the robot, whereas ErrP-classification performance has a direct impact on robot's learning performance.

Different effects of ErrP-classification performance on robot's learning performance between both learning conditions, e.g., the lower number of learning performance of the robot (mapping error) and the lower number of ErrP misclassifications in warm-start learning compared to cold-start learning cannot be explained by our investigation. One could possibly explain it by assuming the following: a subject might eventually have recognized a systematic repetition of wrong assignments of human gesture and robot's action, e.g., *left-right* pairs, the human can expect the upcoming action of the robot (e.g., *right* action) after performing a specific gesture type (*left* gesture) *before* observing the robot's action. We assume that such an expectation of the human would affect the online detection of ErrPs. We further assume that such situations would occur more often in cold-start learning compared to warm-start learning. The chosen algorithm is capable of correcting the wrongly learned gesture-action pairs (relearning). We assumed that more experiences (i.e., more data) are required for relearning (correction of wrong assignments) compared to learning in the initial state (blank state). However, this is a vague interpretation. Thus, the relearning pattern between both learning condition can be investigated in the future to analyze different effects of ErrP-classification performance on learning performance of the robot between both learning conditions.

Further, the descriptive analysis of learning progress in individual datasets (i.e., descriptive visualization of 74 datasets) shows that most subjects showed a stabilization of learning curve after 30 trials (i.e., after adding a new gesture). However, in cold-start learning some subjects seem to require considerably more trials to stabilize the learning curve. This indicates that the time point of adding a new context (gesture) was not optimal for some subjects in cold-start learning. Note that we did not depict all 74 visualizations of learning progress (learning curve) in this paper (just two datasets as examples). We analyzed learning progress by performing inferential statistical analysis, i.e., by statistically comparing mean differences over all subjects between three learning phases (Figure 4), since learning progress of individual datasets can be visualized only descriptively. Note that we visualized 74 learning curves from 74 datasets for each perspective (human's perspective/robot's perspective): 20 datasets × 2 perspectives = 40 datasets; 17 datasets × 2 perspectives = 34 datasets). On the other hand, an outlier can be easily interpreted without explicitly performing an inference statistics as shown in Figure 8 (light green box in the top right side of the visualization). Note that Figure 8 descriptively shows variability between individual datasets in ErrP-classification performance and learning performance of the robot (mapping errors). This outlier (one dataset of one subject) had an extremely low learning performance of the robot and also an extremely low ErrP-classification performance (especially a high number of FN). Actually it is reasonable to understand that the robot could hardly learn correct actions if the ErrP decoder constantly failed to recognize ErrPs. Future investigations should focus on the relationship (correlation) between ErrP-classification performance and learning progress of the robot *per learning phase*, where the determination of learning phase is also a relevant issue for investigations of interaction errors on robot's learning progress.

In general, the number of attempts plays a critical role in reinforcement learning and the agent updates the policy based on rewards that are predefined before learning begins. In HRI, on the other hand, the rewards (human feedback) are generated during online learning and can therefore be influenced by interactions with the robot, i.e., the online learning performance of the robot (e.g., changes in online learning performance during interaction with humans). Therefore, not only the number of attempts but also interaction effects of online learning performance on the generation of human feedback can have an influence on the robot's learning performance (mapping error). Assuming that only the number of trials has an influence on the learning performance of the robot, our results suggest that for some subjects in cold start learning more than 90 trials might be necessary. In practice, it is not always possible to record more than 90 trials from subjects, and recording large numbers of trials is not realistic for some subjects and many applications. One limits oneself to recording a sufficient number of human examples within a reasonable period of time. Indeed, research is needed into the interaction effects between the generation of human feedback and the online learning performance of the robot. It is known that the number of trials (episodes) has an influence on the learning performance of the robot. However, we do not know if increasing the number of trials has a clear effect on the robot's learning performance if there is a human-robot interaction and this interaction influences the generation of EEG-based human feedback. For example, we do not know whether the learning curve stabilizes with the increase in the number of trials (more than 90 tests) for a subject considered an outlier. In our study we did not investigate the effects of the online learning performance of robots on the generation of human feedback, which is very challenging to investigate. As shown in our investigations, the generation of human feedback can also be influenced by other interaction components in HRI (e.g., human gestures). Hence, it is not straightforward to explain subject variability in online learning performance of the robot. In this paper we analyzed the interaction effects of two different interaction components (human feedback and human gesture) on the robot's online learning performance. The question of the interaction effects between the generation of human feedback and the robot's online learning performance, i.e., the effects of the robot's online learning performance on the generation of human feedback, can be investigated in the future.

In most EEG-based BCIs the robot actions were directly corrected binary based on ErrP detections [e.g., *left* (wrong) → *right* (correct) or *right* (wrong) → *left* (correct)] (Salazar-Gomez et al., 2017) or the control policy of robots were learned and optimized based on online ErrP detections (Iturrate et al., 2015; Kim et al., 2017). In a recent study, ErrPs were used for co-adaptation of human and robot (Ehrlich and Cheng, 2018) and for modeling of co-adaptation of human and robot (Ehrlich and Cheng, 2019a). In most studies there was only one interaction component (human feedback, i.e., ErrP) (Iturrate et al., 2015). In our study we have two interaction components (human feedback and human gestures) that can separately or jointly influence the online performance of robots. In this paper, we investigated individual effects of two interaction components on the learning performance of a robot and interaction effects of two interaction components on the learning performance of the robot. Even if learning in a robot is possible without prior knowledge and despite errors in the interpretation of gestures or the detection of ErrP, our results show that it is quite useful to use prior knowledge. They also show that learning with prior knowledge regarding the subjects variability is more stable, which should be investigated more systematically in the future. In general, we could show that errors in both interaction components have less impact on the general learning behavior if previous knowledge is used, whereas false positive results have a greater effect. However, false negative results, i.e., not recognizing mistakes, should be considered more critical. We were able to explain our results partly by the way the learning algorithm used works. However, there are still open questions. For example, the influence of humans is a factor that is difficult to model, but has a great influence on the results. In the future, therefore, the effects of interactions with the robot (changes in the robot's online learning performance) on the online generation of EEG-based human feedback should be analyzed to study the variability of the robot's learning performance depending on the interacting human. Furthermore, our results indicate that both warm start learning (fast convergence) and cold start learning (more exploration) have advantages. For example, it would be possible to give specific prior knowledge (warm start learning) when a change of state is not strongly expected, or to let the agent do natural exploration (cold start learning) to enable the robot to adapt more quickly to likely state changes.

## Data Availability Statement

The datasets presented in this article are not readily available because there is no permission to transfer the data to third parties. Requests to access the datasets should be directed to su-kyoung.kim@dfki.de.

## Ethics Statement

The studies involving human participants were reviewed and approved by the ethics committee of the University of Bremen, Universität Bremen, Rechtsstelle—Referat 06 Bibliothekstraße 1, 28359, Bremen. The patients/participants provided their written informed consent to participate in this study.

## Author Contributions

SK, EK, and FK developed the proposed human-robot interaction (HRI) concept and the HRI scenarios for evaluation of the proposed concept. SK recorded the data and performed data analysis, generated the results including all figures and all tables and performed statistical evaluations, and wrote the methods and results and most parts of introduction and discussion. LS also analyzed the data. EK gave the critical feedback on all parts of sections and wrote introduction and discussion. FK gave the critical feedback on overall works. SK, EK, LS, and FK discussed the results together and wrote the overall manuscript. All authors contributed to the article and approved the submitted version.

## Conflict of Interest

The authors declare that the research was conducted in the absence of any commercial or financial relationships that could be construed as a potential conflict of interest.
