# Supplementary material for: Errors in Human-Robot Interactions and Their Effects on Robot Learning
Source: Front Robot AI. 2020 Oct 15;7:558531. doi: 10.3389/frobt.2020.558531 (PMC7805941; doi:10.3389/frobt.2020.558531)
Supplement: Supplementary file 1 [file Data_Sheet_1.pdf]

Supplementary Material: Errors in human-robot interactions and their effects on robot learning

Su Kyoung Kim [1,*], Elsa Andrea Kirchner [1,2], Lukas Schloßmüller [2], and Frank Kirchner [1,2]
[1]Robotics Innovation Center, German Research Center for Artificial Intelligence (DFKI GmbH), Bremen, Germany
[2]Research Group Robotics, University of Bremen, Bremen, Germany
Correspondence*:
Su Kyoung Kim
su-kyoung.kim@dfki.de

1 SUPPLEMENTARY FIGURES

1.1 Number of datasets for each learning condition and each subject (Tab. S1)

Table S1. Number of datasets for each subject and each learning condition

	warm-start learning	cold-start learning
2 subjects	3 datasets	3 datasets
2 subjects	3 datasets	2 datasets
3 subjects	2 datasets	2 datasets
1 subject	2 datasets	1 dataset
total	20 datasets	17 datasets

1.2 A descriptive visualization of correlation between robot's learning performance (mapping errors) and ErrP-detection performance (FN, FP, TP, TN) for both perspectives (Fig. S1)

1.3 A descriptive visualization of correlation between ErrP-detection performance (FN, FP, TP, TN) and gesture errors for both perspectives (Fig. S2)

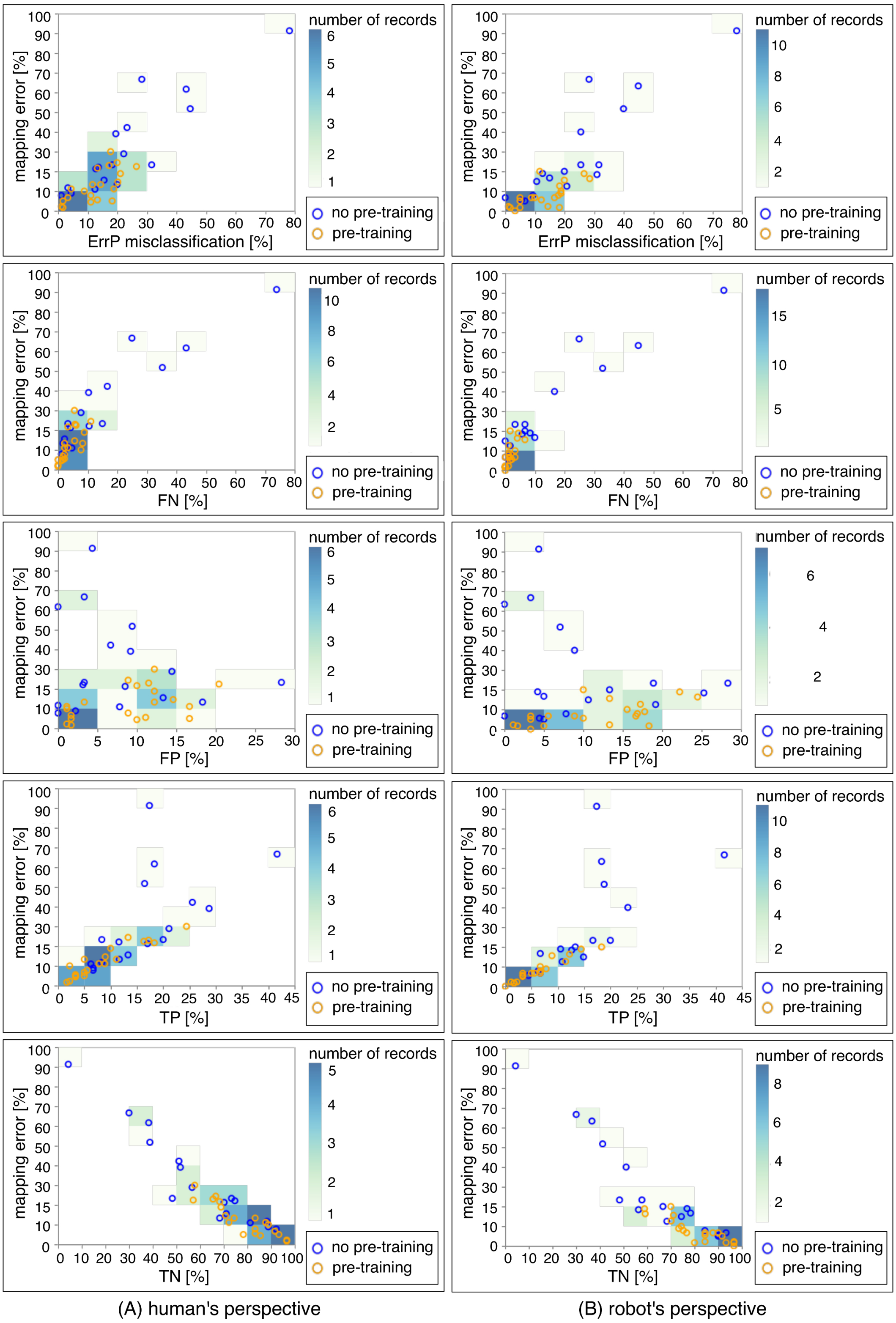

Figure S1. A descriptive visualization of correlation between mapping errors and ErrP-detection performance (FN, FP, TP, TN) for both perspectives: (A) human's perspective and (B) robot's perspective. For each perspective, the comparison between both learning conditions is depicted in different colors: pre-training (yellow) vs. no pre-training (blue). Each dot represents each dataset (details, see text).

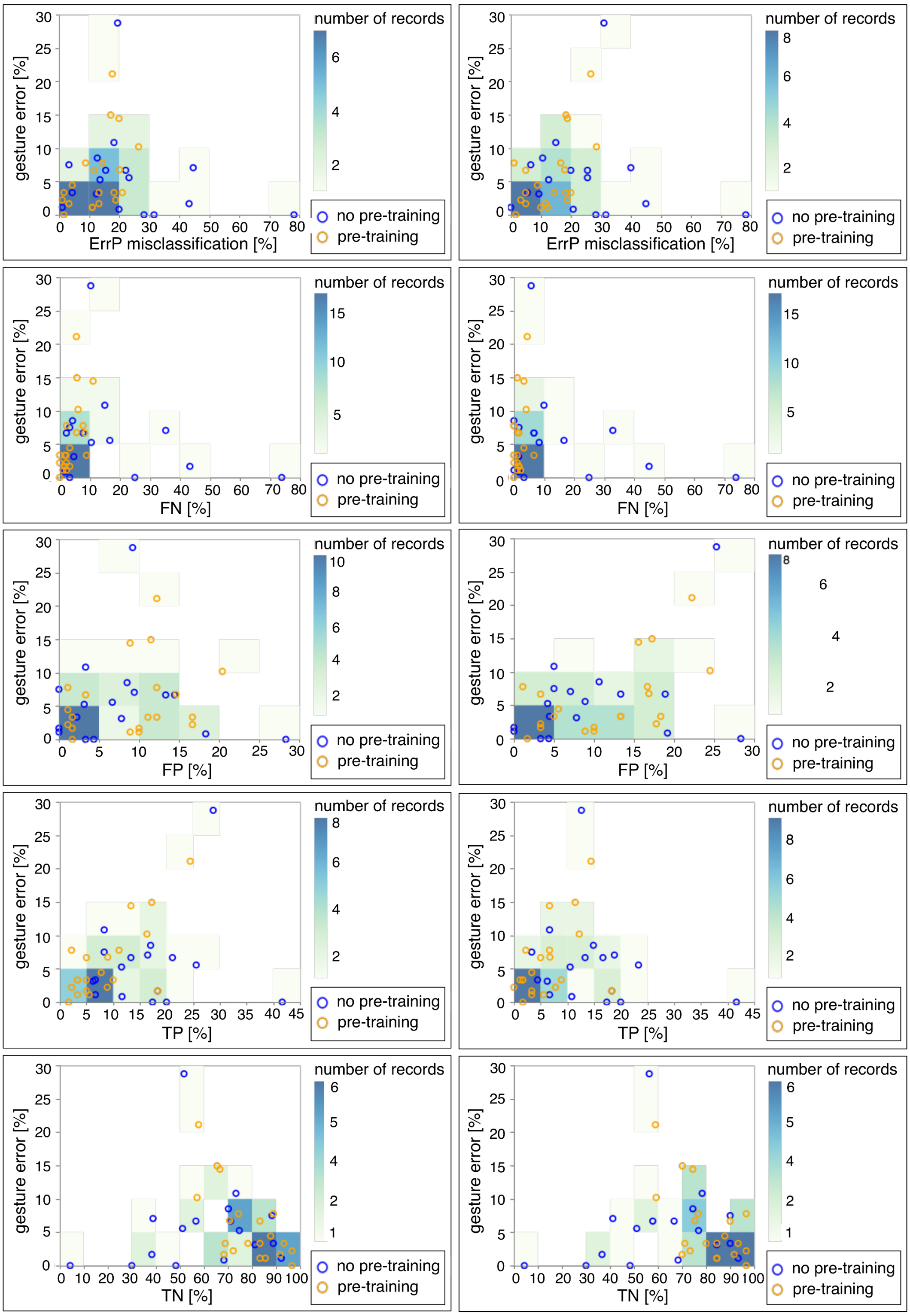

(A) human's perspective

(B) robot's perspective

Figure S2. A descriptive visualization of correlation between gesture errors and ErrP-detection performance (FN, FP, TP, TN) for both perspectives: (A) human's perspective and (B) robot's perspective. For each perspective, the comparison between both learning conditions is depicted in different colors: pre-training (yellow) vs. no pre-training (blue). Each dot represents each dataset (details, see text).
